# Genomic Study of RNA Polymerase II and III SNAP_c_-Bound Promoters Reveals a Gene Transcribed by Both Enzymes and a Broad Use of Common Activators

**DOI:** 10.1371/journal.pgen.1003028

**Published:** 2012-11-15

**Authors:** Nicole James Faresse, Donatella Canella, Viviane Praz, Joëlle Michaud, David Romascano, Nouria Hernandez

**Affiliations:** 1 Center for Integrative Genomics, Faculty of Biology and Medicine, University of Lausanne, Lausanne, Switzerland; 2 Swiss Institute of Bioinformatics, Lausanne, Switzerland; Stanford University School of Medicine, United States of America

## Abstract

SNAP_c_ is one of a few basal transcription factors used by both RNA polymerase (pol) II and pol III. To define the set of active SNAP_c_-dependent promoters in human cells, we have localized genome-wide four SNAP_c_ subunits, GTF2B (TFIIB), BRF2, pol II, and pol III. Among some seventy loci occupied by SNAP_c_ and other factors, including pol II snRNA genes, pol III genes with type 3 promoters, and a few un-annotated loci, most are primarily occupied by either pol II and GTF2B, or pol III and BRF2. A notable exception is the *RPPH1* gene, which is occupied by significant amounts of both polymerases. We show that the large majority of SNAP_c_-dependent promoters recruit POU2F1 and/or ZNF143 on their enhancer region, and a subset also recruits GABP, a factor newly implicated in SNAP_c_-dependent transcription. These activators associate with pol II and III promoters in G1 slightly before the polymerase, and ZNF143 is required for efficient transcription initiation complex assembly. The results characterize a set of genes with unique properties and establish that polymerase specificity is not absolute *in vivo*.

## Author Summary

SNAP_c_-dependent promoters are unique among cellular promoters in being very similar to each other, even though some of them recruit RNA polymerase II and others RNA polymerase III. We have examined all SNAP_c_-bound promoters present in the human genome. We find a surprisingly small number of them, some 70 promoters. Among these, the large majority is bound by either RNA polymerase II or RNA polymerase III, as expected, but one gene hitherto considered an RNA polymerase III gene is also occupied by significant levels of RNA polymerase II. Both RNA polymerase II and RNA polymerase III SNAP_c_-dependent promoters use a largely overlapping set of a few transcription activators, including GABP, a novel factor implicated in snRNA gene transcription.

## Introduction

The human pol II snRNA genes and type 3 pol III genes have the particularity of containing highly similar promoters, composed of a distal sequence element (DSE) that enhances transcription and a proximal sequence element (PSE) required for basal transcription. In pol II snRNA promoters, the PSE is the sole essential core promoter element whereas in type 3 pol III promoters, there is in addition a TATA box, which determines RNA pol III specificity [1], [2]. The PSE recruits the five-subunit complex SNAP_c_, one of the few basal factors involved in both pol II and pol III transcription. Basal transcription from pol II snRNA promoters requires, in addition, TBP, TFIIA, GTF2B (TFIIB), TFIIF, and TFIIE, and from pol III type 3 promoters TBP, BDP1, and a specialized GTF2B-related factor known as BRF2 [3], [4], [5]. The DSE is often composed of an octamer and a ZNF143 motif (Z-motif) that recruit the factors POU2F1 (Oct-1) and ZNF143 (hStaf), respectively [1], [2]. POU2F1 activates transcription in part by binding cooperatively with SNAP_c_ and thus stabilizing the transcription initiation complex on the DNA (see [6], and references therein).

In addition to requiring some different basal transcription factors for transcription initiation, pol II and pol III transcription at SNAP_c_-recruiting promoters differ in the way transcription terminates. In pol III genes, there are runs of T residues at various distances downstream of the RNA-coding sequence, which direct transcription termination ([7] and references therein). In pol II snRNA genes, a “3′ box” starting generally 5–20 base pairs downstream of the RNA coding sequence directs processing of the RNA, with transcription termination reported to occur either just downstream of the 3′ box [8], or over a region of several hundreds of base pairs [9].

Although model snRNA promoters have been extensively studied, it is unclear how broadly SNAP_c_ is used, and to what extent the highly similar pol II and pol III PSE-containing promoters are selective in their recruitment of the polymerase. It is also unclear how generally the use of the basal factor SNAP_c_ is coupled to that of the activators POU2F1 and ZNF143, and by which mechanisms ZNF143 activates transcription. To address these questions, we performed genome-wide immunoprecipitations followed by deep sequencing (ChIP-seq) to localize four of the five SNAP_c_ subunits, GTF2B, BRF2, and a subunit of each pol II and pol III. These studies define a set of SNAP_c_-dependent transcription units and show that although most loci are primarily bound by one or the other polymerase, the *RPPH1* (RNase P RNA) gene is occupied by both enzymes. Pol II is detectable up to 1.2 kb downstream of the end of the RNA-coding regions of pol II snRNA genes, thus defining a broad region of transcription termination. Localization of POU2F1 and ZNF143 shows widespread usage of these activators by PSE-containing promoters, and we find that several of these promoters also bind the activator GABP [10], which has not been implicated in snRNA gene transcription before. Activators are recruited before the polymerase in G1, and this process is less efficient when ZNF143 levels are decreased by RNAi.

## Results

### Identification of genes occupied by SNAP_c_ and RNA polymerase

We performed ChIP-seq with antibodies against SNAPC4 (SNAPC190), the largest SNAP_c_ subunit, SNAPC1 (SNAP43), and SNAPC5 (SNAP19) in IMR90Tert cells. To localize SNAPC2 (SNAP45), we used an IMR90Tert cell line expressing both biotin ligase and SNAPC2 tagged with the biotin acceptor domain for chromatin affinity purification (ChAP)-seq (see [11]). We also used antibodies against GTF2B, which should mark pol II snRNA promoters, BRF2, which should mark type 3 pol III promoters, and POLR2B (RPB2), the second largest subunit of pol II. We used POLR3D (RPC4) ChIP-seq data [11] to localize pol III.

Most of the human pol II snRNA and type 3 pol III genes are repeated and/or have given rise to large amounts of related sequences within the genome. We therefore aligned tags as described before [11], excluding tags aligning with one or more mismatches but including tags with several perfect matches in the genome (see Methods). We selected regions containing at least two SNAP_c_ subunits and either BRF2 and pol III, or GTF2B and pol II, as described in Methods. We obtained loci encompassing all known type 3 pol III genes as well as most annotated pol II snRNA genes. In addition, we obtained a few novel loci occupied by SNAP_c_ and pol II. Table S1 shows these loci as well as the annotated snRNA genes that did not display any tags, namely four *RNU1* and one *RNU2* snRNA genes (in red in the first column). It also shows, in grey, *RNU2* genes that are still in the “chr17_random” file of the human assembly and were thus not in the reference genome used for tag alignment.

In some cases, we noticed adjacent POLR2B peaks separated by only one or a few nucleotides, which often corresponded to annotated SNP positions. Inclusion of tags aligned with ELAND, which allows for some mismatches, often resulted in the fusion of adjacent peaks, as for the *SNORD13* gene shown in Figure S1A (compare upper and lower panels). Such loci are likely to be occupied by POLR2B –indeed their promoter regions are occupied by significant amounts of GTF2B and SNAP_c_ subunits– and they are labeled in yellow in the first column of Table S1. In a few cases, however, this did not result in fusions of adjacent peaks, as shown in Figure S1B for a *RNU1* gene (*U1-12*). Such peaks probably result from attribution of tags with multiple genomic matches to an incorrect genomic location and are thus likely to be artifacts. Consistent with this possibility, *U1-11*, *U1-12*, *U1-like-8*, *U3-2*, *U3-2b*, *U3-4*, and *U3-3*, all labeled in orange in Table S1, had POLR2B, GTF2B, and SNAP_c_ subunits scores with either 0% or, in the cases of *U3-4*, less than 15%, unique tags. We consider these loci unlikely to be occupied by pol II *in vivo*. In contrast, the POLR2B peak on the *RNU2* snRNA gene on chromosome (chr) 11, even though interrupted about 500 base pairs downstream of the snRNA coding region, is constituted mostly of unique tags, as are the GTF2B and SNAP_c_ subunit peaks. This gene is likely, therefore, to be indeed occupied by pol II and other factors, and is labeled in striped yellow in the first column (Table S1).

### Pol II and pol III genes occupied by SNAP_c_

**Figure 1:**
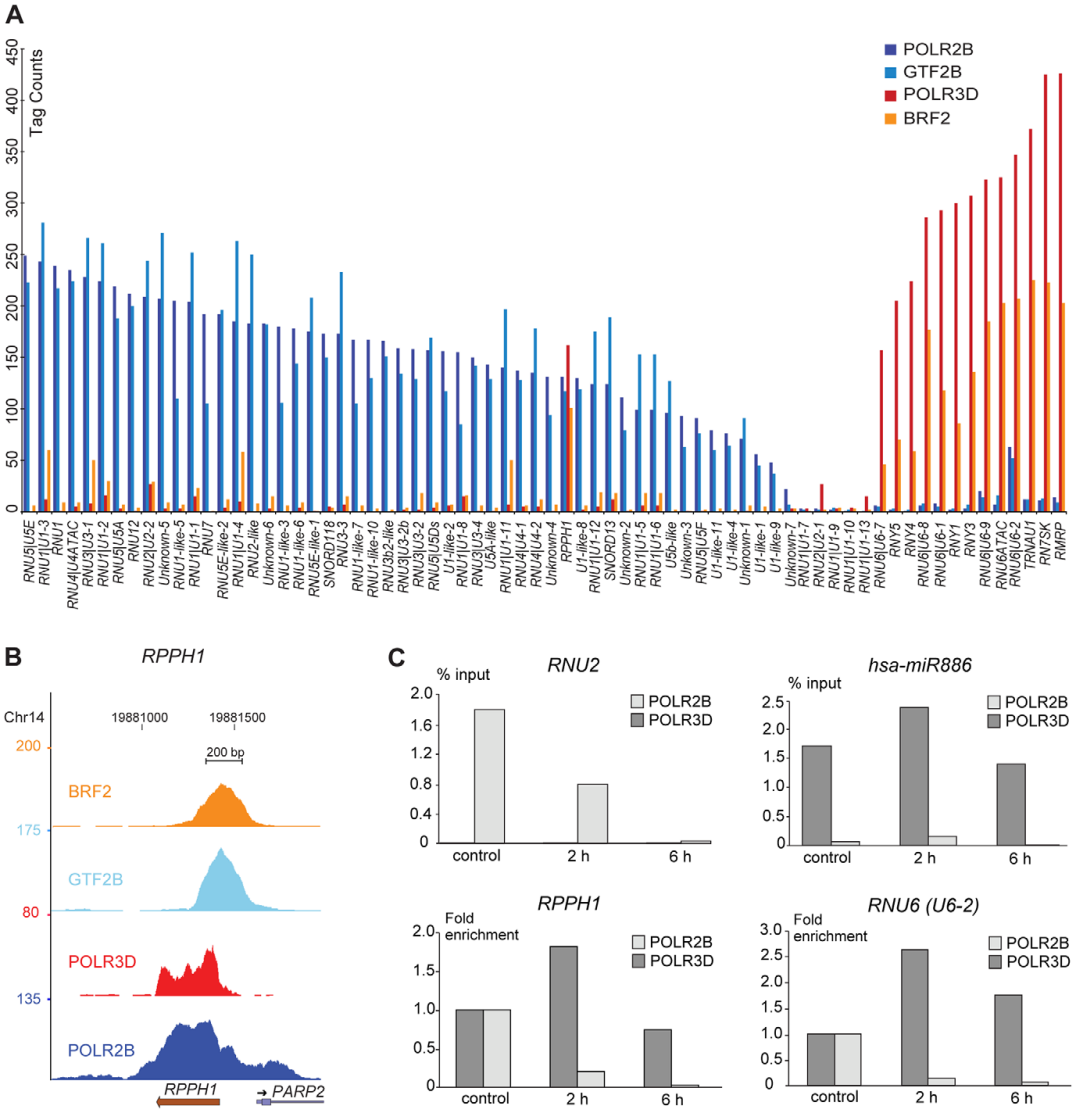
Pol II and III occupancy of snRNA genes. (A) Bar graph showing POLR2B (dark blue), GTF2B (light blue), POLR3D (red), and BRF2 (orange) ChIP-seq scores (y axis) on SNAP_c_-occupied genes and the few snRNA genes devoid of SNAP_c_ (x axis). Genes are ordered by decreasing POLR2B scores for the pol II and *RPPH1* genes followed by increasing POLR3D scores for the pol III genes. (B) UCSC browser view of *RPPH1* gene showing POLR2B, POLR3D, GTF2B, and BRF2 occupancy. Y axis: tag counts. (C) POLR2B (light grey) or POLR3D (dark grey) occupancy in cells not treated or treated with 50 µg/ml α-amanitin for 2 or 6 h, as indicated on the x axis. Upper two panels: results are shown as % of input. Lower two panels: POLR2B and POLR3D occupancy without α-amanitin was set at 1.

We calculated occupancy scores for all loci by adding tags covering peak regions, as described in Methods (see legend to Table S1 for exact regions). We first examined the POLR2B, POLR3D, GTF2B, and BRF2 scores. For most genes there was a clear dominance of either POLR2B and GTF2B or POLR3D and BRF2 (Figure 1A). Further, there was a good correlation between POLR2B and GTF2B (0.89) or POLR3D and BRF2 (0.80) scores, but not between POLR2B and BRF2 (0.075), or POLR3D and GTF2B (0.22) (Figure S2). This is consistent with GTF2B and BRF2 being specifically dedicated to recruitment of pol II and pol III, respectively, and indicates that most SNAP_c_-occupied genes are transcribed primarily by a single polymerase.

Strikingly, among SNAP_c_-occupied promoters, only thirteen loci were occupied primarily by BRF2 and pol III (listed on top of Table S1), corresponding to the known type 3 genes previously shown to be occupied by pol III in IMR90hTert and other cell lines [11], [12], [13], [14]. We identified a larger number of SNAP_c_-bound loci occupied primarily by GTF2B and pol II. They included genes coding for the U1, U2, U4 and U5 snRNAs, all involved in splicing of pre-mRNAs; U11, U12, and U4atac snRNAs, which have similar functions as U1, U2, and U4 but participate in the removal of a smaller class of introns referred to as AT-AC introns; U7 snRNA, involved in the maturation of histone pre-mRNAs; U3, U8, and U13 small nucleolar RNAs (snoRNAs), involved in the maturation of pre-ribosomal RNA, as well as snRNA-derived sequences. The relationship of these loci with previously described snRNAs and snoRNA genes is described in the Results section of Text S1. We also uncovered a few non-annotated loci harboring SNAP_c_ subunits, as well as GTF2B and POLR2B, peaks constituted by at least 20% of unique tags and, therefore, likely to correspond to new actively transcribed regions. These are labeled *Unknown-1* to *7* (rows 76–82 in Table S1). As described below, these sequences harbor a PSE as well as some other sequence elements typical of pol II snRNA promoters, and contain similarities to the 3′ box.

### *RPPH1* is occupied by BRF2 and POLR3D as well as by GTF2B and POLR2B

Although most genes were occupied mostly by either BRF2 and POLR3D, or GTF2B, and POLR2B, there were a few exceptions. The most notable was the *RPPH1* gene, which is considered a type 3 pol III gene [15] but was in fact occupied not only by BRF2 and POLR3D but also by significant amounts of POLR2B and GTF2B, comparable to those found on the *RNU4* snRNA genes (Figure 1A and 1B). This suggested that this gene could be transcribed *in vivo* by either of two RNA polymerases, pol II or pol III. To explore this possibility further, we treated cells with a concentration of α-amanitin known to inhibit pol II but not pol III transcription [16]. As expected, this treatment reduced the POLR2B signal of the pol II *RNU2* gene but not the POLR3D signal on the pol III *hsa-mi-886* gene (Figure 1C, upper panels). To determine the effects of α-amanitin for the *RPPH1* gene and the *U6-2* gene, which also displayed some POLR2B signal in addition to the expected POLR3D signal (see Figure 1A), we set the POLR2B and POLR3D signals obtained in the absence of α-amanitin at 1. In each case, addition of α-amanitin to the medium reduced the POLR2B but not the POLR3D signal (Figure 1C, lower panels). Thus, the *RPPH1* gene can be transcribed either by pol II or pol III *in vivo*.

### Location of SNAP_c_ subunits GTF2B and BRF2 on pol II and III promoters

**Figure 2:**
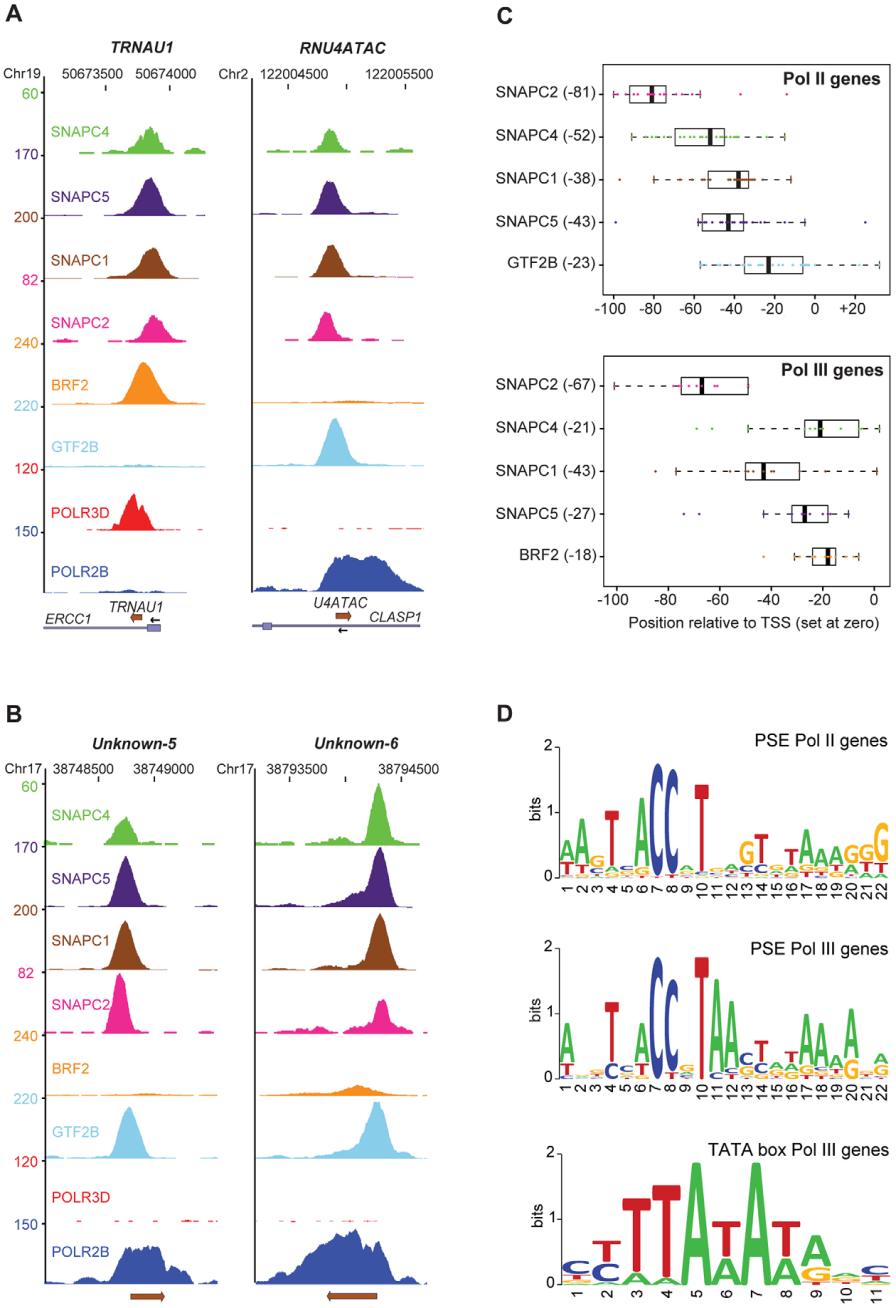
SNAP_c_ subunits occupancy and proximal promoter motifs. (A) UCSC browser views of a pol III (*tRNAU1*) and a pol II (*RNU4atac*) gene showing occupancy by the factors indicated on the left. The chromosome coordinates are shown on top, the genes present in the region and their orientation at bottom. The y axis shows tag counts. (B) Two examples of non-annotated genomic regions showing occupancy by SNAP_c_ subunits, GTF2B, and POLR2B. (C) Box plot of BRF2, GTF2B, and SNAP_c_ subunit positions. For each gene, the position of the peak summit for each SNAP_c_ subunit relative to the TSS (set at 0) was determined. A median position (black bars in boxes, number in brackets on the y axis) was calculated. For the pol II genes, only the upper two tertiles of each SNAP_c_ subunit and GTF2B scores were included. The position for each gene is represented by a circle. (D) LOGOs of PSE and TATA box generated by WebLogo with the motifs identified with MEME (alignments in Figures S4 and S5). The top panel shows the PSE LOGO for pol II snRNA genes, the middle panel shows the PSE LOGO for pol III genes, and the bottom panel shows the TATA box LOGO for pol III genes.

One of the criteria used to select the genes in Table S1 was the presence of at least two of the four SNAP_c_ subunits examined. We obtained a good correlation between scores for the four SNAP_c_ subunits tested (Figure S3), consistent with SNAP_c_ binding as a single complex to snRNA promoters [17]. Figure 2A shows the peaks obtained for the SNAP_c_ subunits, BRF2, GTF2B, POLR3D, and POLR2B on the pol III *TRNAU1* gene and the pol II *RNU4ATAC* gene, and Figure 2B shows two non-annotated genomic loci occupied by POLR2B, GTF2B, and SNAP_c_ subunits. Whereas the polymerase subunits were detected over the entire RNA coding sequence of the corresponding genes (and further downstream in the case of POLR2B), the other factors were located within the 5′ flanking region, with GTF2B and BRF2 close to, or overlapping, the TSS. Although peaks were sometimes constituted of too few tags to allow an unambiguous determination of the peak summit location (see for example the SNAPC4 peak in Figure 2A), we could nevertheless detect clear trends. The GTF2B or BRF2 peaks were generally the closest to the TSS, the SNAPC4, SNAPC1, and SNAPC5 peaks were within the PSE sequence, and the SNAPC2 peak was upstream of the PSE (Figure 2C).

Figure S4 shows an alignment of the PSEs and TATA boxes of the 14 pol III type 3 promoters (including the *RPPH1* gene), and Figure S5 an alignment of the PSEs of all pol II loci listed in Table S1. The non-annotated loci occupied by POLR2B and factors contain clear PSEs. Moreover, as noted previously [1], [2], the PSE is located further upstream of the TSS in pol III than in pol II snRNA genes. The corresponding LOGOs revealed similar but not identical consensus sequences for the PSEs of pol II and pol III genes (Figure 2D); for example, adenines were favored in positions 11 and 12 of pol III, but not pol II, PSEs. Thus, although the TATA box is the dominant element specifying RNA polymerase specificity –indeed the U2 and U6 PSEs can be interchanged with no effect on RNA polymerase recruitment specificity [16]– the exact PSE sequence may also contribute to specific recruitment, for example in the context of a weak TATA box.

### Pol II terminates transcription within the 1.5 kb downstream of mature snRNA–coding sequences

**Figure 3:**
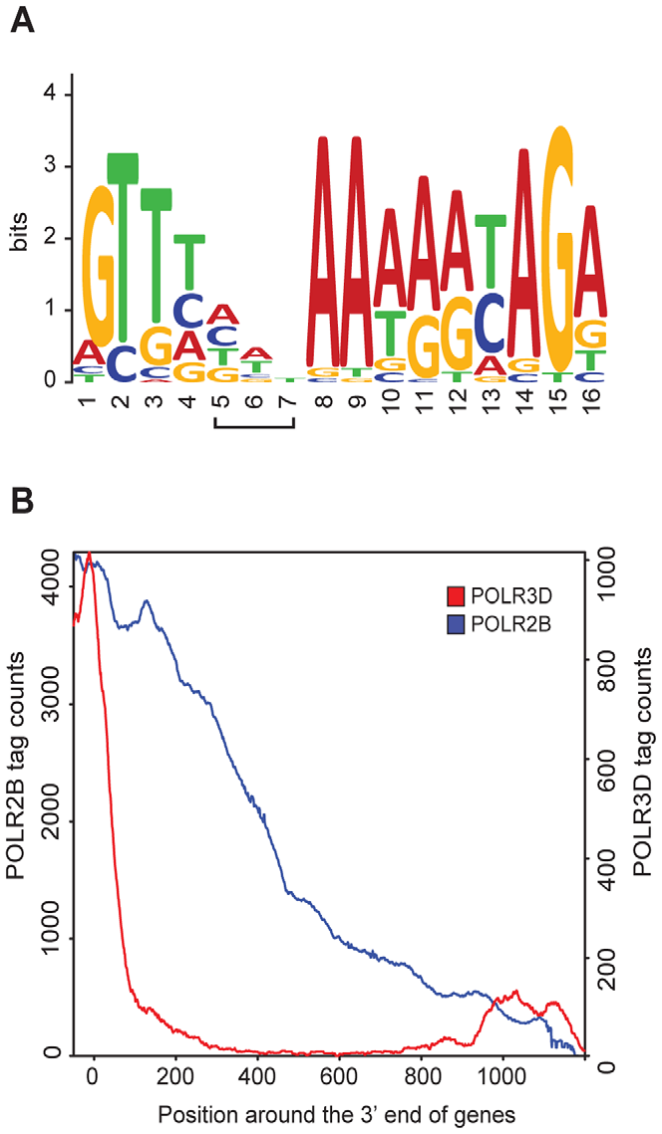
RNA pol II and III occupancy within 3′ flanking regions. (A) 3′ box LOGO generated by WebLogo with the motifs found within 100 bp downstream of the RNA coding sequence of Pol II genes (see alignment in Figure S6). The bracketed positions 5–7 of the LOGO correspond to the positions that are sometimes gaps in the alignment of Figure S6. (B) Graphical representation of POLR2B (in blue) and POLR3D (in red) tag accumulation past the 3′ end of the RNA-coding region of pol II and pol III genes, respectively. X axis: position around the 3′ end of the RNA coding regions (set at 0). Y axes: tag counts for POLR2B on the left and POLR3D on the right.

The U1 and U2 snRNA genes are followed by a processing signal known as the 3′ box [18], [19], which is also found downstream of several other pol II snRNA genes [1]. We could identify 3′ boxes in most of the pol II genes in Table S1. An alignment of these motifs allowed us to generate a matrix with GLAM2 [20], which we then used to search for 3′ boxes in all pol II with GLAM2SCAN [20]. As shown in Figure S6, we could identify putative 3′ boxes downstream of all annotated pol II genes in Table S1 (except for the non-expressed *RNU1 (U1-9)* and *RNU1 (U1-13)* genes), as well as for the non-annotated genes. For the *RPPH1* gene, the best match to a 3′ box was located within the RNA coding sequence, from −73 to −61 relative to the end of the RNA coding sequence (Figure S6). The resulting 3′ box LOGO derived from all sequences aligned in Figure S6 is shown in Figure 3A.

**Figure 4:**
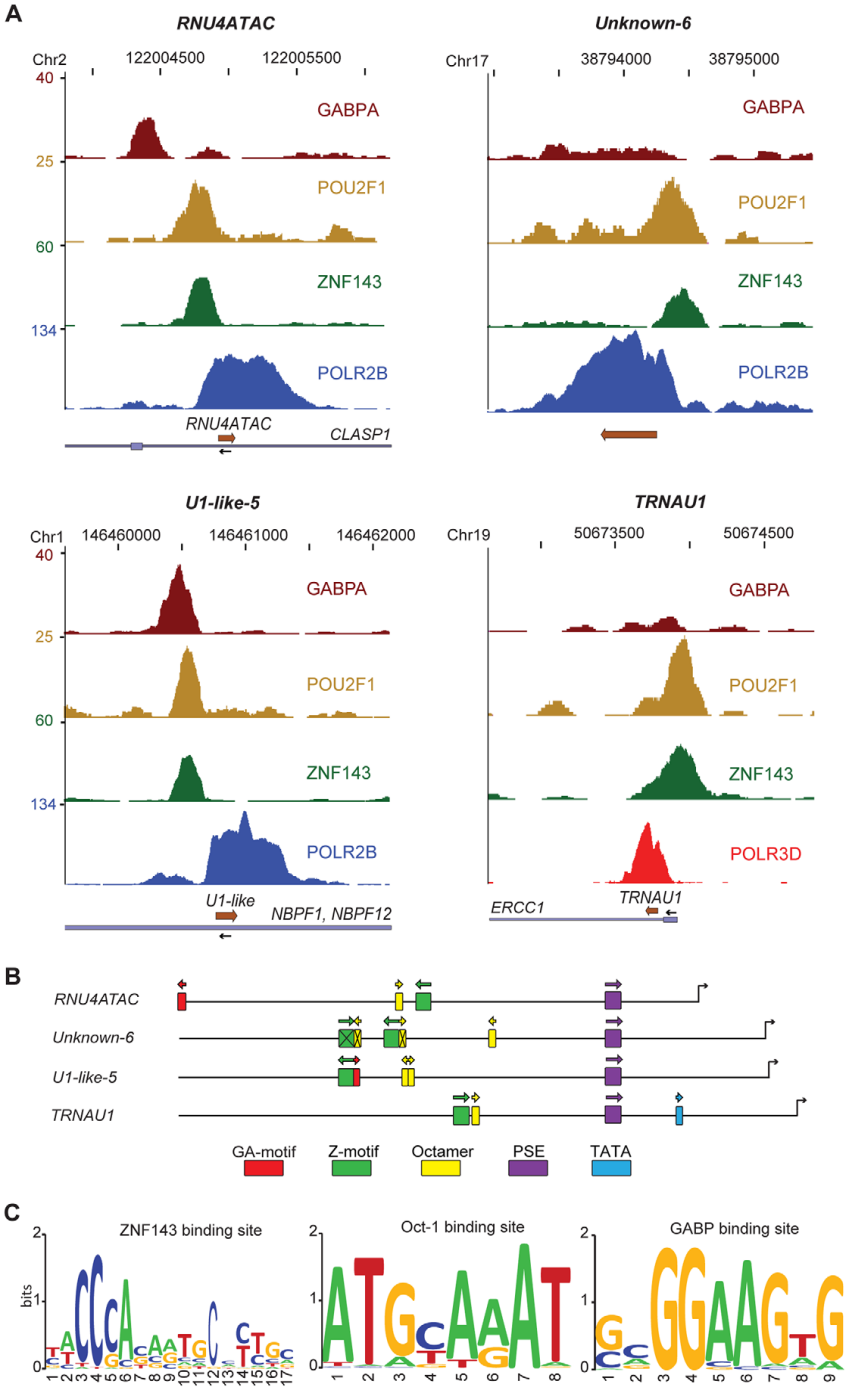
Activator occupancy and distal promoter motifs. (A) UCSC browser view of three pol II (*RNU4atac*, *U1-like-5*, and *Unknown-6*) and one pol III (*tRNAU1*) gene showing occupancy by the factors indicated on the right of each panel. The chromosome coordinates are shown on top, the genes present in the region and their orientation at bottom. The y axis shows tag counts. (B) Promoter region (−400 to +1) of the four genes depicted in (A) with the positions of the GABPA (GA-motif), ZNF143 (SBS), and POU2F1 (octamer) binding sites found by MEME or MAST indicated. The positions of the PSE and TATA box are also shown, and the promoters were aligned according to the PSE position. The crossed-out motifs have either no corresponding peak of occupancy or are not the closest to the peak summit. The orientation of each motif is indicated with an arrow. (C) LOGOs of the ZNF143, POU2F1 (octamer) and GABP binding motifs generated by WebLogo with the motifs located closest to the corresponding factor peak summits (see alignments in Figures S9, S10, S11).

Pol II transcription termination has been reported to occur either shortly after, or several hundred base pairs downstream of, the 3′ box [8], [9]. Our POLR2B ChIP-seq data reveal the extent of pol II occupancy downstream of the RNA coding region. Whereas on average, the POLR3D ChIP-seq signal dropped quite abruptly downstream of the RNA coding region of pol III genes (see [7]), POLR2B could be detected as far as about 1200 base pairs past the RNA coding region of pol II snRNA genes (Figure 3B). Moreover, examination of the POLR2B peak downstream of individual pol II genes revealed a gradual decrease of tag counts over regions of 500 or more base pairs (see for example Figure 2A and 2B, and Figure 4A below). Thus, transcription termination occurs well downstream of the 3′ box and over a broad region.

### The POU2F1, ZNF143, and GABP proteins are often bound to SNAP_c_-recruiting promoters

snRNA promoters are characterized by an enhancer element (DSE) typically containing an octamer motif and a ZNF143 binding site (Z-motif), which in some specific genes has been shown to recruit, respectively, the POU domain protein POU2F1 and the zinc finger protein ZNF143 (see [1], [2] and references therein). To determine how general the binding of POU2F1 and ZNF143 is among SNAP_c_-binding promoters, we localized POU2F1 by ChIP-seq in HeLa cells and we analyzed ChIP-seq data obtained by others in HeLa cells (JM, VP, and Winship Herr, personal communication) for ZNF143 and, as ZNF143 was found to bind often together with GABP (JM, VP, and Winship Herr, personal communication), for the α subunit of GABP (GABPA). The scores for all genes are listed in Table S1 and, in a summarized form, in Table S2. The pol III genes in Table S1, which were all occupied by basal factors (see above), were each occupied by at least one activator. Among pol II genes, those not occupied by basal factors (labeled in red in the first column of Tables S1 and S2) did not display peaks for any of the activators, and those with interrupted POLR2B peaks (orange in the first column) had peaks composed solely of tags with multiple matches in the genome, consistent with the possibility raised above that these genes are, in fact, not occupied by factors.

Of the genes clearly occupied by basal factors, all displayed peaks for at least one activator with three exceptions, *U1-like-11*, *unknown-2,* and *unknown-3*; these last three loci had basal factor peaks with relatively low scores and thus may bind some of these activators at levels too low to be detectable in our analysis. Most genes had a POU2F1 peak (93%), a large majority had a ZNF143peak (81%), and about half had a GABPA peak (45%). Interestingly, some genes had specific combinations of activators; for example the *RNU5* and *U5-like* genes as well as most pol III genes had peaks for both POU2F1 and ZNF143 but not for GABPA. In contrast *RNU6ATAC*, *SNORD13*, and *RNU3* genes had POU2F1 and GABPA peaks but no ZNF143 peak. Only few genes had only one activator (*RMRP*, *RNY4*, *RNU2-2*, *U3b2-like*, *RNU7*, and *Unknown-5*) suggesting that most snRNA genes require some combination of the three activators tested for efficient transcription. Indeed, altogether 23 genes had peaks for all three factors and 23 had peaks for both ZNF143 and POU2F1 but not GABPA. Thus, the very large majority (79%) of SNAP_c_-binding genes bound both POU2F1 and ZNF143. The scores for the various activators were surprisingly correlated (see Figure S7), perhaps indicating that these factors bind to snRNA promoters interdependently. Figure 4A shows two examples (*RNU4ATAC* and *U1-like-5*) with the three factors present, and two examples (*Unknown-6* and *tRNAU1*) with only POU2F1 and ZNF143. In all cases, the factors bound upstream of the PSE with GABP, when present, generally binding the furthest upstream.

We analyzed 5′ flanking sequences for motifs and identified POU2F1 (octamer, see [21]), ZNF143 [22], [23], and GABP [24], [25], [26] binding sites (Figure 4B, Figure S8A and S8B). This analysis revealed a high concordance between occupancy as determined by ChIP-seq and presence of the corresponding motif, with only a few cases (GABP and ZNF143 for *U1-like-10*, and GABP for *U5E-like*, *U4-1*, and *unknown-7* genes) where no convincing motif could be identified. We then aligned all occupied motifs (see Figures S9, S10, and S11) to generate the LOGOs shown in Figure 4C, which thus reflect the ZNF143, POU2F1, and GABP binding sites in SNAP_c_-recruiting genes.

### Basal factors as well as activators are recruited to the U1, U2, and U6 snRNA promoters upon transcription activation in G1

**Figure 5:**
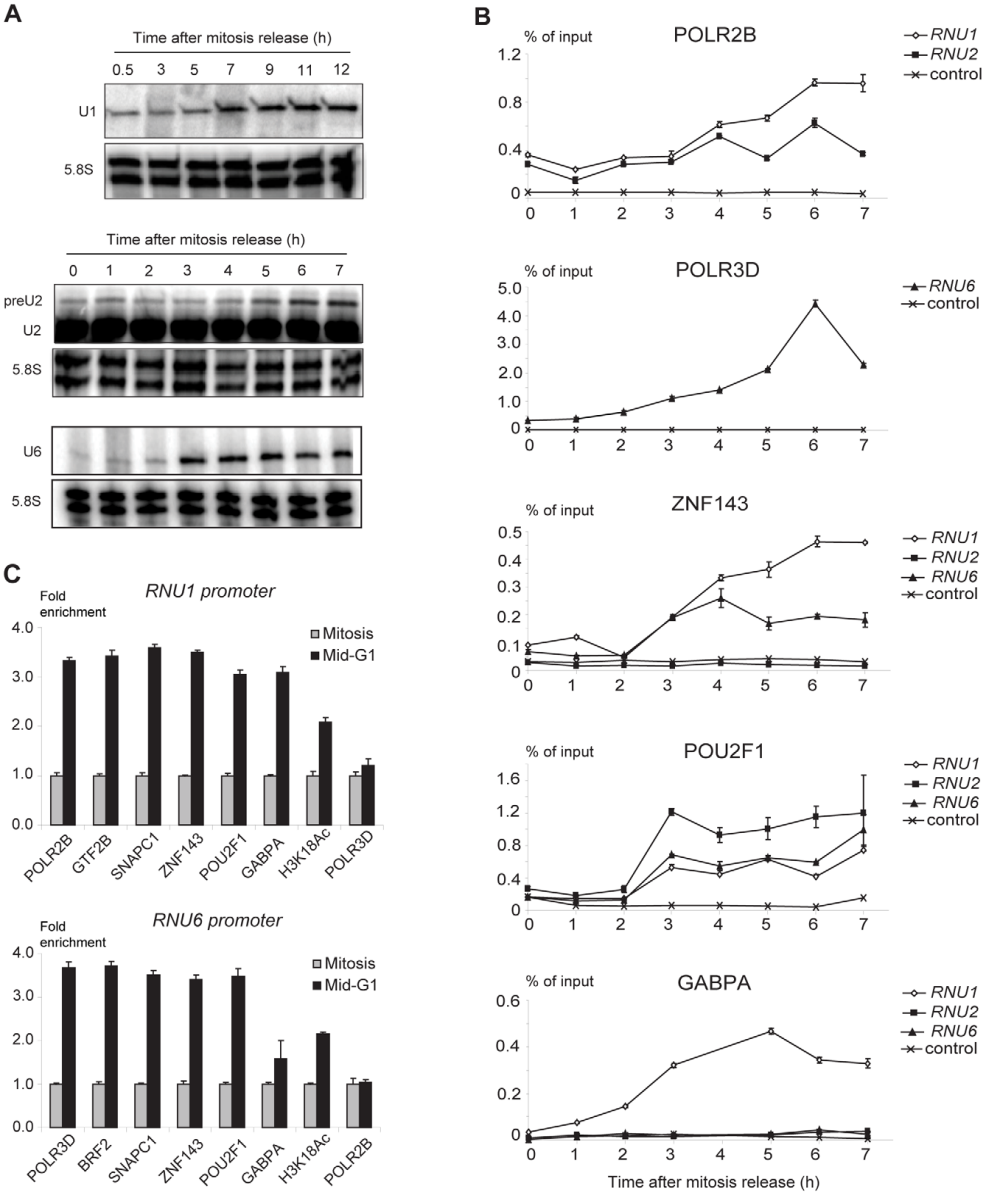
*RNU1*, *RNU2*, and *RNU6* transcription and factor recruitment during mitosis to G1 phase transition. (A) Time course of U1 and U6 reporter transcript and U2 and pre-U2 snRNA accumulation after mitosis release. The 5.8S RNA served as an internal control. The time after mitosis release is indicated above each panel. (B) Time course analysis of transcription factor recruitment on various promoter regions. ChIPs were performed at the times indicated (x axis) after mitosis release with antibodies directed against the factors indicated on top of each panel, and analyzed by real time PCR. The analyzed regions are indicated at the upper right of each panel. The control region (Ctrl) is 2 kb upstream of *RNU1*. The results are expressed relative to input DNA. Two sets of *RNU1* primers were used: set U1A recognizes *U1-1, U1-2, U1-3, U1-8, U1-like-3* loci and was used in the top panel; set U1B recognizes *U1-2* and *U1-3* loci and was used in the 3 lower panels. The *RNU2* primers are specific for the *RNU2* cluster in chr17_unknown, and the *RNU6* primers for the *U6-1* locus. (C) Real time PCR analysis of *RNU1* (top panel, U1A primer set for POLR2B, GTF2B, SNAPC1 and POLR3D ChIPs; and U1B primer set for the other ChIPs) and *RNU6* (bottom panel) promoters pulled down after ChIP with antibodies against the factors indicated below the panels either at mitosis (1 h after release) or in mid-G1 (7 h after release). The results are expressed relative to mitosis values, which were set at 1 for each factor. Means and error bars were calculated over triplicate PCR analyses. Each experiment was performed at least twice.

Transcription of *RNU6* and probably *RNU1* and *RNU2* is known to be low during mitosis and to increase as cells cycle through the G1 phase [27], [28], [29], [30], [31], hence we measured the levels of U1, U2, and U6 snRNA during mitosis and at several times after entry into G1. Since snRNA transcripts are very stable, making it difficult to measure transcription variability, we generated HeLa cell lines containing *RNU1* or *RNU6* reporter construct expressing unstable transcripts whose levels therefore better reflect ongoing transcription. For U2 snRNA, we measured its precursor, which has a short half-life [16]. Cells were blocked in prometaphase with Nocodazole and released with fresh medium. RNA levels were low during mitosis and, in the case of the U1 reporter RNA and pre-U2 RNA, increased to a maximum 6–7 h after release, around the middle of the G1 phase (as determined by FACS analysis, see Methods). For the U6 reporter RNA, RNA levels reached a maximum 3 h after release, at the beginning of the G1 phase (Figure 5A). POLR2B occupancy was apparent 4 h after the mitosis release and peaked after 6 h, as measured by ChIP-qPCR analysis of both *RNU1* and *RNU2* loci (Figure 5B). This was specific, as no significant amounts of POL2RB were detected on the control region. In comparison, increased POLR3D occupancy of *RNU6* (but not the control region) was apparent 3 h after release and peaked after 6 h, consistent with the accumulation of U6 RNA earlier in G1 than U1 and U2 RNA.

We then examined promoter occupancy by transcription activators (Figure 5B). ZNF143 occupancy increased over time on both the *RNU1* and *RNU6* promoters, becoming clearly detectable at 3 h and reaching a maximum at 6 h for *RNU1* and 4 h for *RNU6*. In contrast, ZNF143 was undetectable on the *RNU2* promoters. POU2F became detectable at 3 h on the *RNU1*, *RNU2*, and *RNU6* promoters and then remained at a more or less constant level. GABP was detected only on the *RNU1* promoters and was recruited early, starting 2 h after the release and reaching a maximum at 5 h. Thus, activators were recruited on the promoters expected from the ChIP-seq data above, with kinetics slightly faster than the polymerase. Among activators, GABP was recruited the earliest, followed by concomitant recruitment of ZNF143 and POU2F1.

Some basal transcription factors such as TBP are thought to remain bound to chromatin, and hence probably promoters, during mitosis [32], [33]. To explore whether this is the case for SNAP_c_, GTF2B, and BRF2, we monitored occupancy by these factors at mitosis (1 h after release) and in mid-G1 (7 h after release). On the pol II *RNU1* snRNA promoter, we observed enrichment of GTF2B and SNAP_c_ subunits, as well as the pol II subunit POLR2B, the activators ZNF143, POU2F1, and GABP, and H3 acetylated on lysine 18 (H3K18Ac) at mid-G1 compared to mitosis (Figure 5C, upper panel). This was specific as the pol III subunit POLR3D was not enriched. On the pol III *RNU6* promoter, we observed enrichment of POLR3D, BRF2, SNAP_c_ subunits, ZNF143, POU2F1 and H3K18Ac, but not POLR2B nor GABP, as expected (Figure 5C, lower panel). This suggests that at snRNA promoters, both basal transcription factors and activators are removed from promoter DNA during mitosis and are recruited de novo upon transcription activation in G1.

### ZNF143 is essential for factor recruitment to a pol II and a pol III snRNA promoter

**Figure 6:**
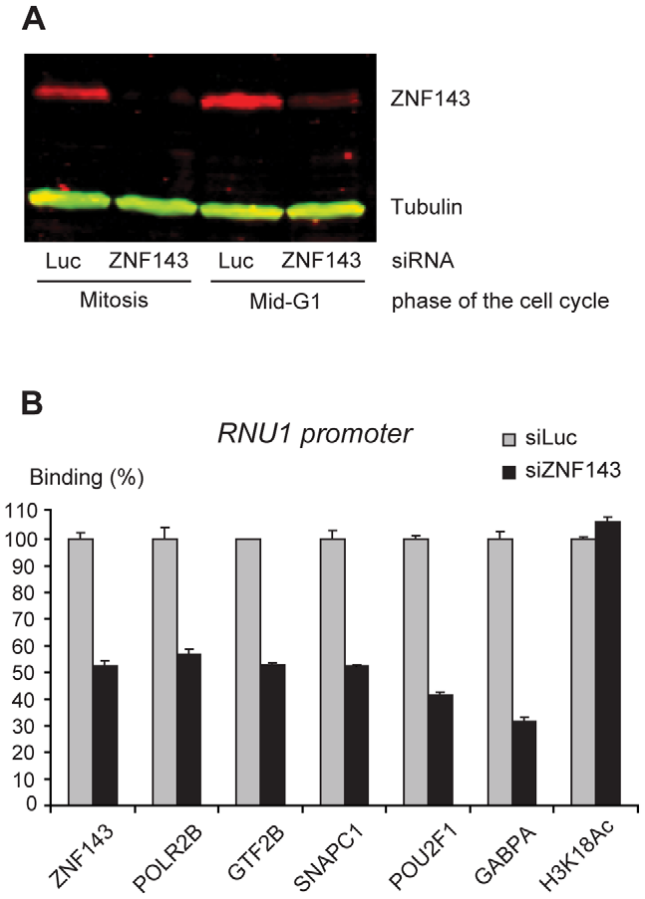
Depletion of endogenous ZNF143 reduces transcription factor recruitment on the U1 promoter in mid-G1. (A) Immunoblot showing ZNF143 and Tubulin (control) levels during mitosis and mid-G1 phase after treatment with siRNA against Luciferase (Luc, control siRNA) or ZNF143. (B) Real time PCR analysis of *RNU1* promoter pulled down after ChIP with antibodies against the factors indicated below the panel either after treatment of the cells with siRNA against Luciferase (siLuc, control siRNA) or siRNA against ZNF143 (siZNF143). The values obtained with the siZNF143 treatment are shown relative to those obtained with the siLuc treatment, which were set at 100%. Means and error bars were calculated over triplicate PCR analyses. Each experiment was performed at least twice. The U1A primer set was used for the POLR2B, GTF2B and SNAPC1 ChIPs, the U1B primer set for the other ChIPs.

To explore the role of ZNF143 in transcription factor recruitment, we targeted endogenous ZNF143 by siRNA and synchronized the cells as above. Total protein levels measured both at mitosis and in mid-G1 were reduced by more than 70% (Figure 6A), and in mid-G1, ZNF143 bound to the U1 promoter was decreased by 50% (Figure 5B). Under these conditions, binding of the activators POU2F1 and GABP, the basal transcription factors GTF2B and SNAPC1, and POL2RB were reduced by 40 to 70%. In contrast, the H3K18Ac levels were not reduced (Figure 6B). Thus, ZNF143 contributes to efficient recruitment of other activators, basal transcription factors, and the RNA polymerase, but not to H3K18 acetylation, at the pol II U1 promoter.

## Discussion

Using stringent criteria of co-occupancy by two SNAP_c_ subunits and either GTF2B and pol II, or BRF2 and pol III, we identified a surprisingly small number of SNAP_c_-occupied promoters comprising the 14 known type 3 pol III promoters, some 40 pol II snRNA genes, and 7 novel pol II-occupied loci. It seems, therefore, that in cultured cells, SNAP_c_ is a very specialized factor participating in the assembly of transcription initiation complexes at fewer than 100 promoters. We have not explored, however, the possibility that some of the SNAP_c_ subunits participate in transcription of other genes or in other functions as part of complexes other than SNAP_c_. Indeed, in a previous localization of SNAP_c_ subunits on genomic sites also binding TBP, a correlation analysis on non-CpG islands split the SNAP_c_ subunits into two subgroups, one containing SNAPC1 and SNAPC5 and the other SNAPC2, SNAPC3, and SNAPC4 [34], consistent with the possibility that other SNAP -subunit-containing complexes exist.

A peculiarity of SNAP_c_ is its involvement in transcription from both pol II and pol III promoters, promoters that differ from each other mainly by the presence or absence of a TATA box. We found that most SNAP_c_-occupied promoters were predominantly occupied by either pol II or pol III with two exceptions, the *U6-2* and most notably the *RPPH1* genes, which were occupied not only by BRF2 and pol III, as expected, but also by levels of GTF2B and pol II comparable, in the second case, to those found on some pol II snRNA genes. We showed that pol II occupancy of the *RPPH1* gene was obliterated by levels of α-amanitin shown before to inhibit pol II transcription in cultured cells [16]. Previous experiments comparing the 3′ ends of pol II and pol III transcripts derived from wild-type and mutated versions of the human *RNU2* and *RNU6* promoters have shown that pol II-synthesized transcripts end downstream of a signal referred to as the “3′ box” whereas pol III-synthesized transcripts are not processed at such boxes and instead end at runs of T residues [16]. The best similarity to a 3′ box lies within the *RPPH1* RNA coding region. However, we detect only one type of transcript, terminated at the run of T residues downstream of the *RPPH1* gene, in endogenous RNA from proliferating IMR90Tert cells (data not shown), suggesting that the transcript synthesized by pol II is highly unstable, at least under the conditions tested. It is conceivable that the ratio of *RPPH1* genes transcribed by pol II and pol III, as well as the ratio of stable pol II and pol III RNA products, change in different cell types or under different conditions. The observation that a gene can be transcribed by two different polymerase *in vivo* thus raises the possibility of an added layer of complexity in the regulation of gene expression. It is not clear why the *U6-2* and *RPPH1* promoters are capable of recruiting significant levels of pol II. The *RPPH1* promoter has a short TATA box, but the *U6-7* and *U6-8* promoters have the same TATA box and are not promiscuous. An intriguing possibility is that the presence of a 3′ box at a correct distance downstream of the TSS, together with a weak TATA box, allow pol II recruitment.

The locations of the occupancy peaks for the four SNAP_c_ subunits we tested are remarkably consistent with what is known about the architecture and DNA binding of SNAP_c_. SNAPC4, the largest SNAP_c_ subunit and the backbone of the complex, binds directly to the PSE through Myb repeats located in the N-terminal half of the protein [35]. SNAPC1 and SNAPC5 associate directly with SNAPC4, N-terminal of the Myb repeats (aa 84–133, see [36]). Consistent with this architecture, we find that SNAPC4, SNAPC1, and SNAPC5 generally peak very close to each other within the PSE. In contrast, SNAPC2, which associates with the C-terminal part of SNAPC4 (aa 1281–1393, see [36]), peaks upstream of the PSE. This suggests that the N-terminus of SNAPC4 is oriented facing the transcription start site whereas the C-terminal part is oriented towards the upstream promoter region. This is consistent with the orientation of D. melanogaster SNAPC4 [37] on the U1 and U6 D. melanogaster snRNA promoters as determined by elegant studies combining site-specific protein-DNA crosslinking with site-specific chemical protein cleavage ([38], see also [39] and references therein).

The 3′ end of pol II snRNAs is generated by processing at a sequence called the 3′ box [2], [40]. The 3′ box is efficiently used only by transcription complexes derived from snRNA promoters, suggesting that the polymerase II recruited on these promoters is somehow different from that recruited on mRNA promoters. Indeed, the C-terminal domain of pol II associated with snRNA genes carries a unique serine 7 phosphorylation mark, which recruits RPAP2, a serine 5 phosphatase, as well as the integrator complex, both of which are required for processing ([41] and references therein; [42], [43]). Moreover, pol II transcription of snRNA genes requires a specialized elongation complex known as the Little Elongation Complex (LEC) [44]. It has been unclear, however, how far downstream of the 3′ box processing signal transcription continues, with one report indicating a very sharp drop in transcription within 60 base pairs past the U1 3′ box [8] and another reporting continued transcription for several hundreds of base pairs downstream of the U2 3′ box [9]. Our ChIP-seq data indicate that pol II can be found associated with the template more than 1 Kb downstream of the 3′ box, for both the *RNU1* and *RNU2* genes as well as all other pol II snRNA genes. This suggests that transcription termination downstream of snRNA gene 3′ boxes does not occur at a precise location but rather over a broad 1.2 Kb region, and is triggered by passage of the polymerase through the processing signal, reminiscent of transcription termination downstream of the poly A signal, in this case in a region of several Kbs [45].

Activation of several SNAP_c_-dependent promoters has been shown to depend on a DSE and on the binding of POU2F1 and ZNF143 (see [1], [2] and references therein, [23]). Our ChIP-seq analyses show that POU2F1 and ZNF143 are associated with the large majority of SNAP_c_-dependent promoters and identify GABP as a new factor binding to a subset of these promoters. During transcription activation in G1, we observed binding of ZNF143 and POU2F1 preceding binding of RNA pol II and pol III, consistent with the possibility that binding of these activators prepares the promoters for polymerase recruitment. Indeed, lowering the amount of ZNF143 by siRNA strongly affected recruitment of POU2F1, GABPA, basal factors, and the polymerase itself on the U1 promoter. Thus, ZNF143 could either recruit and stabilize POU2F1 by direct protein-protein contact, or affect chromatin structure to allow recruitment of POU2F1, or both. In support of the first hypothesis, ZFP143, the mouse homolog of ZNF143, recruits another POU-domain protein, Oct4 (the mouse homolog of POU5F1) by direct association [46]. On the other hand, ZNF143 and POU2F1 do not bind cooperatively to the human *U6-1* promoter [47], but then *U6-1* is weakly POLR3D-occupied compared to other human RNU6 genes [11]. In support of the second possibility, we have shown before that ZNF143 can bind to an snRNA promoter, in this case the pol III U6 snRNA promoter, preassembled into chromatin [48], suggesting that it is an early player in the establishment of a transcription initiation complex. However, promoter H3K18 acetylation, which is low just after mitosis and increases during G1, was unaffected. This suggests that SNAP_c_-dependent promoters are targeted very early in G1 by as yet unidentified factors that lead to histone modifications, in particular H3K18 acetylation. It will be interesting to determine how this modification combines with the H3K4me3 mark observed on pol III promoters, including type 3 pol III promoters [12], [13], [14], [49].

## Methods

### ChIPs

ChIPs were performed as described [11]. The antibodies used (rabbit polyclonal antibodies except where indicated) were as follows: POLR3D, CS682, directed against the C-terminal 14 aa [50]; POLR2B, H-201 from Santa Cruz Biotechnology; BRF2, 940.505 #74; GTF2B, CS369 #10, 11; SNAPC4, CS696 #4,5; SNAPC5, CS539 #7,8; SNAPC1, CS47 #7,8; GABP, sc-22810 X from Santa Cruz Biotechnology; POU2F1, mix of YL8 and YL15 [51], [52] or mix of two polyclonal antibodies (A310-610A from Bethyl Laboratories); ZNF143, antibody 19164 raised against ZNF143 aa 623–638, [48]. The ChAPs have been described [11].

### Analysis

The sequence tags obtained after ultra-high throughput sequencing were mapped onto the UCSC genome version Hg18, corresponding to NCBI 36.2, as before [11] except that we included tags mapping to up to 500 rather than 1000 different locations in the genome. Table S3 shows the total number of tags sequenced for each ChIP and the percentages of tags mapped onto the genome. In all cases, 75.5% or more of the total tags mapped onto the genome had unique genomic matches.

Peaks were detected with sissrs (www.rajajothi.com/sissrs/) [53] with a false discovery rate set at 0.001%, as previously described [11]. We identified 77312 POLR2B, 4838 GTF2B, 1366 POLR3D, and 2526 BRF2 peaks. We then selected the POLR2B peaks within 100 base pairs of a GTF2B peak (3878 peaks), and the POLR3D peaks within 100 base pairs of a BRF2 peak (125 peaks). The ChIPs with the anti-SNAP_c_ subunit antibodies gave relatively weak signals. We therefore divided the genome into 200 nucleotide bins, counted tags obtained for each of the four SNAP_c_ subunits analyzed, and retained only bins displaying an enrichment for at least two of the SNAP_c_ subunits. Bins were considered positive only if the tag number in bin reached at least the minimum tag count determined by sissrs for enriched regions with a 0.001 false discovery rate as the one used in sissrs set at the default parameters. We then considered genomic regions containing POLR2B and GTF2B, or POLR3D and BRF2, sissrs peaks as well as a bin positive for two SNAP_c_ subunits within 100 nucleotides of the polymerase sissrs peak. We obtained 157 and 58 loci for the POLR2B and POLR3D lists, respectively, which were all visually inspected. We eliminated peaks in regions of high background, with shapes never found in known snRNA genes (for example peaks with rectangular shapes resulting from artefactual accumulation of tags), or with identical shape and location in all samples. The most convincingly occupied loci are listed in Table S1, which also shows all annotated pol II snRNA genes, whether or not they were found occupied by POLR2B, GTF2B, and SNAP_c_ subunits. Scores were calculated as described in [49] and contained a component consisting of the sum of tags with unique matches in the genome and another representing tags with multiple matches in the genome: such tags were attributed a weight corresponding to the number of times they were sequenced divided by the number of matches in the genome, with a maximum weight set at 1. In Table S1, the score percentage contributed by unique tags is indicated in separate columns. Scores and peak shapes are more reliable for scores consisting mostly of unique tags, as in these cases there is no ambiguity as to where in the genome tags should be aligned.

For the SNAP_c_ subunits, we confirmed the results of the first analysis by performing a second analysis in which we counted tags in 200 nucleotide bins as before, then fitted a normal distribution to the data, and used the normal distribution's standard deviation and mean to attribute a P-value for each SNAP_c_ subunit to each genomic bin. We then adjusted it with Benjamini & Hochberg (BH) correction and kept the bins with an adjusted P-value under 0.005 that were located within a 100 nucleotides of either a RPB2 and TF2B positive region, or a RPC4 and BRF2 positive region (as defined by sissrs). We then applied a second filter to keep only the bins containing at least two (of the four mapped) SNAP_c_ subunits. This gave us a total of 275 bins, which contained all the genes listed in Table S1 except for 10 loci. Of these 10 loci, 5 of them are flagged Table S1 as being not occupied (U1-7, U1-9, U1-10, U1-13, U2-1). The remaining five (U1-like-1, U1-like-11, RNU5 (U5F), UNKNOWN-2, and RNU6-7 (U6-7)) have low scores. The additional regions with positive bins (93 regions) corresponded to regions of high background and were eliminated after visual inspection.

### Transient transfections, cell lines, synchronization

To measure *RPPH1*-dependent transcription in vivo, 1.2×10^6^ HeLa cells were transiently transfected (48 hours) with pU6/Hae/RA.2 [16] or derivatives containing the wild-type *RPPH1* promoter, or the *RPPH1* promoter harboring a mutation in the TATA box (TTATAA changed to TCGAGA), as well as the *RPPH1* 3′ flanking region. To specifically inhibit POLR2B transcription, the cells were treated with 50 µg/ml of α-amanitin (Santa Cruz Biotechnology, sc-202440) for two or six hours before harvesting.

Clonal cell lines expressing U1 or U6-promoter-directed unstable RNA were established by transfection of HeLa cells with plasmid derivatives of pU6/RA.2+U6end-Dsred [48] (see Methods section of Text S1 for details). Individual clones were expanded and tested for expression of the U1 or U6 construct. HeLa cell lines were synchronized as described [54]. Briefly, cells were first incubated for 24 h with 2 mM of Thymidine, then 3 h with normal medium, then 14 h with 0.1 mg/ml of Nocodazole. Cells were then harvested (M phase) or transferred to normal medium and harvested at different time points. The cell cycle stage of each sample was determined by flow cytometry analysis with the UV precise T kit (Partec, Germany), which involves isolation of nuclei followed by DAPI staining.

### RNAse T1 protection, siRNA treatments

RNA was extracted from HeLa cells with TRIzol reagent (Invitrogen) according to the manufacturer's protocol and analyzed by RNase T1 protection as before (see Methods section of Text S1 for details). To reduce levels of endogenous ZNF143, a siRNA duplex was generated (Microsynth) to target the ATAAGCTGTGGTACCATCTTCCAGCTG region of the ZNF143 gene. HeLa cells were seeded at 2×10^6^ cells per 10 cm plate the day before transfection. Thirty µl of INTERFERin transfection reagent (Polyplus) was added to 1 ml of DMEM serum-free medium containing 60 nM of siRNA duplex, incubated for 15 minutes, and added to the 10 cm plate containing 10 ml of medium. As negative control, we used a siRNA directed against the firefly luciferase [55] (Dharmacon). Two other siRNA treatments were performed 12 and 24 h after the first transfection. Thirty hours after the 1^st^ transfection, the cells were synchronized as described above.

### Data access

The data can be accessed at NCBI Gene expression Omnibus (http://www.ncbi.nlm.nih.gov/geo) under accession number GSE38303.

## Supporting Information

Figure S1 Interrupted peaks and SNPs. (A). UCSC genome browser view of the *SNORD13* (U13) gene showing the RPB2 peaks obtained when excluding (upper panel) or including (lower panel) tags aligning with mismatches (as selected by the ELAND software) onto the reference genome. (B). As in (A), but for the *RNU1* (U1-12) genomic region.
(TIF)

Figure S2 Spearman correlations of scores for genes occupied by pol II, pol III, GTF2B, and BRF2. The scores obtained for the indicated factors refer to all genes listed in Table S1 (except for the *RNU2* genes in chr17_random).
(TIF)

Figure S3 Spearman correlations of scores for genes occupied by all SNAPc subunits tested (SNAPC1, SNAPC2, SNAPC4, SNAPC5). The scores obtained for the indicated factors refer to all genes listed in Table S1 (except for the *RNU2* genes in chr17_random).
(TIF)

Figure S4 Alignment of pol III PSEs and TATA boxes. The 5′ flanking sequence of the indicated pol III genes is displayed up to position -1. The PSE and TATA box regions are indicated with a thick line, with the PSE and TATA box as defined in [40] in bold. The numbers refer to the first and last position of the sequences under the thick lines relative to the +1 TSS position. The *RNU6* genes are numbered as in [56]. Note that the *RPPH1* sequence contains many SNPs.
(DOC)

Figure S5 Alignment of pol II PSEs. The 5′ flanking sequence of the indicated pol II genes is displayed up to position -1. The *RPPH1* gene is also displayed. The PSE region is indicated by the thick line with the PSE as defined in [40] in bold. The numbers refer to the first and last position of the sequences under the thick line relative to the +1 TSS position. Note that the following sequences are identical in the region shown: *U3-1* and *U3-3; U3-2, U3-2b*, and *U3-4; U1-2, U1-3*, and *U1-4*; *U1-5* and *U1-6*; *U1-11* and *U1-12*.
(DOC)

Figure S6 Alignment of 3′ boxes. Sequences resembling 3′ boxes (consensus sequence GTTT N_1–4_ AANA^A^/_G_ N AGA, see [40]) within the 100 nt following the RNA-coding sequence (+1 to +100, with the 3′ end of the RNA coding region set at 1) were identified manually. These motifs were used to generate a matrix with GLAM2 [20] (which allows gaps), which was then used to search for motifs in all sequences with GLAM2SCAN [20]. The GLAM2SCAN analysis confirmed all motifs except the two shown in italics, and identified motifs in the novel un-annotated genes as well as some additional motifs (underlined). In the *RPPH1* gene, the best match to a 3′ box was found inside the RNA coding sequence.
(DOC)

Figure S7 Spearman correlations of scores for genes occupied by ZNF143, POU2F1, and GABP. The scores obtained for the indicated factors refer to all genes listed in Table S1 (except for the *RNU2* genes in chr17_random).
(TIF)

Figure S8 Schematic representation of promoter regions. (A) For each pol II gene in Table S1 (except for the *RNU2* genes in chr17_random) as well as the *RPPH1* gene, the different motifs found in the promoter region (from −400 to +1 relative to the TSS, except for *U2-like*, which has a GA motif from −1172 to −1164 upstream of the TSS) are represented by colored boxes as indicated. The direction of the motifs (as shown in the alignments in Figures S9, S10, and S11) is indicated with an arrow. The motifs that appeared not occupied, either because there was no corresponding ChIP-Seq occupancy peak or because they were not closest to the occupancy peak summit, are shown crossed-out (black crosses). In some cases, as for example in the divergent octamers in *U1-like-5*, two motifs appeared as likely to be occupied. The promoters are aligned relative to the PSEs and ranked by the POLR2B scores. (B) As in (A), but for the pol III genes in Table S1. The grey box indicates a motif (consensus GNC(^T^/_A_)G (^C^/_G_)(^G^/_C_)NN(^C^/_G_)(^C^/_T_)(^C^/_A_)(^C^/_G_)(^G^/_C_)CG(^G^/_C_)(^G^/_A_)G) of unknown function found in nearly all type 3 pol III genes. The genes are aligned relative to the TATA box and ranked by the POLR3D scores.
(TIF)

Figure S9 Alignment of octamer sequences. Sequences similar to the POU2F1 binding site (octamer) located within peaks of POU2F1 occupancy in the 5′ flanking regions of the indicated genes, except for the octamers in *RNY4*, *U1-9, U1-13*, and *unknown-3*, which are not occupied. In *unknown-6*, the second octamer closer to the TSS is the best centered under the peak summit even though it is a less good octamer than the one further upstream. The numbers refer to the first and last position of the sequences shown relative to the +1 TSS position. The genes not shown in the list (*U1-7, U1-10*, and *U1-13*) have no matches (with up to two mismatches) to the octamer up to 400 bp upstream of the TSS. All octamer sequences present in the alignment are shown as boxes in Figure S8A and S8B. Sequences labeled with an asterisk are the closest to the POU2F1 peak summit on the corresponding promoter, and were used to generate the octamer LOGO shown in Figure 4C. The *U1-like-1/-5/-6/-7/-8/-9* promoter regions have two overlapping octamers of similar quality located in each case near the POU2F1 peak summit; one or both of these motifs may be occupied.
(PDF)

Figure S10 Alignment of Z-motifs. Sequences similar to ZNF143 binding sites (Z-motif) located within peaks of ZNF143 occupancy in the 5′ flanking regions of the indicated genes, except for the Z-motif in the *RMRP* gene and the more upstream Z-motif in the *RPPH1* gene, which are not under a ZNF143 peak. The numbers refer to the first and last position of the sequences shown relative to the +1 TSS position. The sequences in bold were identified by a MAST [20] search with the consensus Z-motif [25] or a similar motif identified by a MEME [20] *de novo* search of motifs present under ZNF143 peaks. All Z-motifs present in the alignment are shown as boxes in Figure S8A and S8B. Sequences labeled with an asterisk are the closest to the ZNF143 peak summit on the corresponding promoter, and were used to generate the ZNF143 binding site LOGO shown in Figure 4C.
(PDF)

Figure S11 Alignment of GA-motifs. Sequences similar to GABP binding sites (GA-motif) present within 400 bp upstream of the TSSs of the genes in Table S1 (except for the *RNU2* genes in chr17_random) identified by MAST [20] with the GABP consensus sequence [25] are indicated in bold, a few additional ones found manually (located under GABP peaks of occupancy) are indicated in standard font. All GA-motifs present in the alignment are shown as boxes in Figure S8A and S8B. The sequences located within peaks of GABP occupancy and closest to the peak summit are indicated with an asterisk and were used to generate the GABP binding site LOGO in Figure 4C. The numbers refer to the first and last position of the sequences shown relative to the +1 TSS position. For some *U1-like* genes, two GABP sites of similar quality were identified under the GABP peak.
(PDF)

Table S1 List of genomic loci occupied by at least two SNAP_c_ subunits and either POLR2B and GTF2B, or POLR3D and BRF2 and all UCSC annotated snRNA gene (whether or not occupied by factors) together with the occupancy scores for POLR2B, GTF2B, POLR3D, BRF2, SNAPC5, SNAPC1, SNAPC4, SNAPC2, ZNF143, POU2F1, and GABPA.
(XLS)

Table S2 Summary of the occupancy scores for ZNF143, POU2F1, and GABPA for the same genomic loci as in Table S1.
(XLS)

Table S3 Number of tags with unique and multiple matches mapped onto the genome for each ChIP_Seq experiment.
(PDF)

Text S1 Results section describing the relationship between loci listed in Tables S1 and S2 and previously studied pol II snRNA and snoRNA genes. Methods section providing details about the stable cell lines used and about the RNase T1 protection assay. References section.
(DOC)
