# Supplementary figures and images for: Genomic Study of RNA Polymerase II and III SNAPc-Bound Promoters Reveals a Gene Transcribed by Both Enzymes and a Broad Use of Common Activators
Source: PLoS Genet. 2012 Nov 15;8(11):e1003028. doi: 10.1371/journal.pgen.1003028 (PMC3499247; doi:10.1371/journal.pgen.1003028)

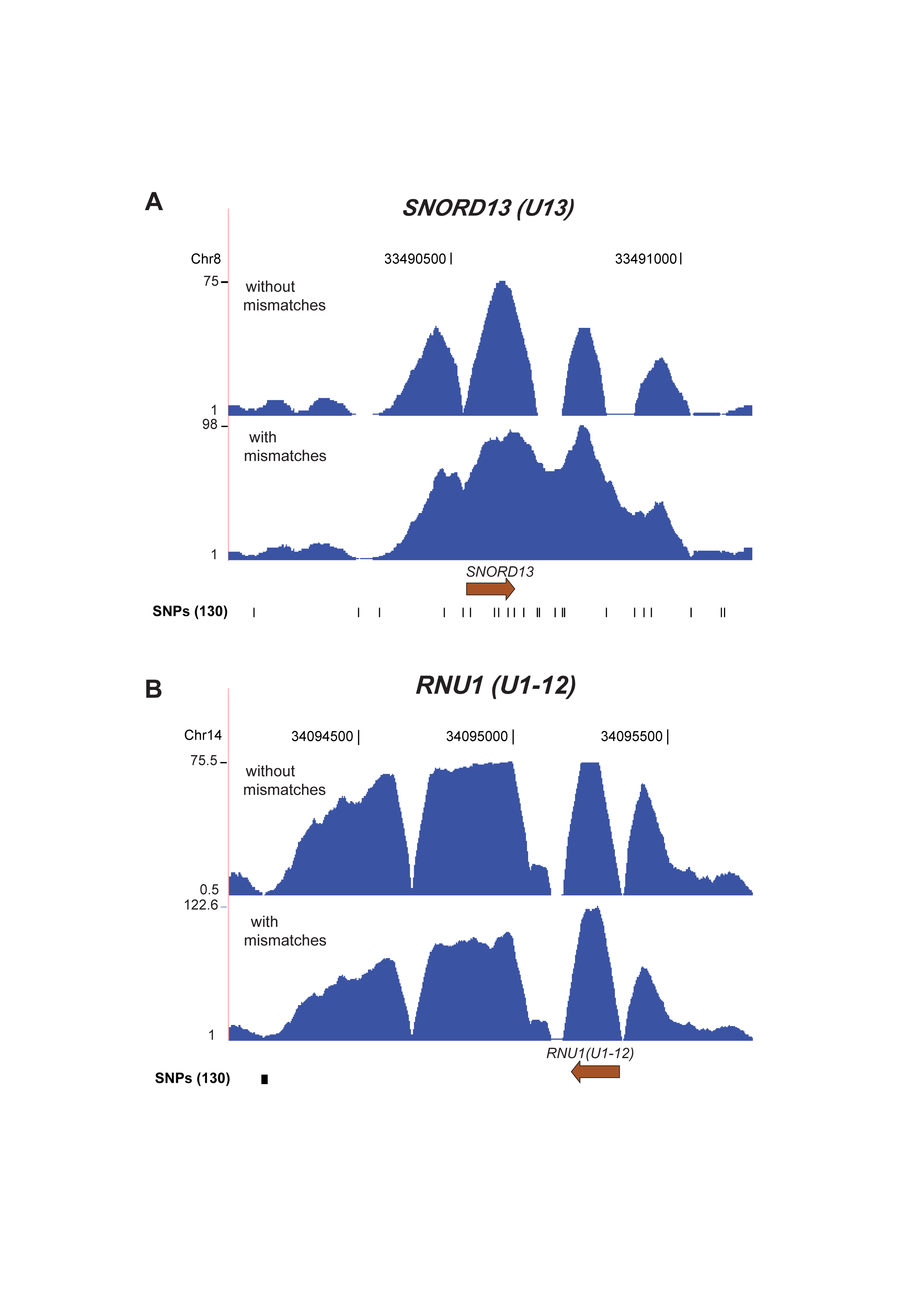

Supplement: Figure S1 — Interrupted peaks and SNPs. (A). UCSC genome browser view of the SNORD13 (U13) gene showing the RPB2 peaks obtained when excluding (upper panel) or including (lower panel) tags aligning with mismatches (as selected by the ELAND software) onto the reference genome. (B). As in (A), but for the RNU1 (U1-12) genomic region. (TIF) [file pgen.1003028.s001.tif]

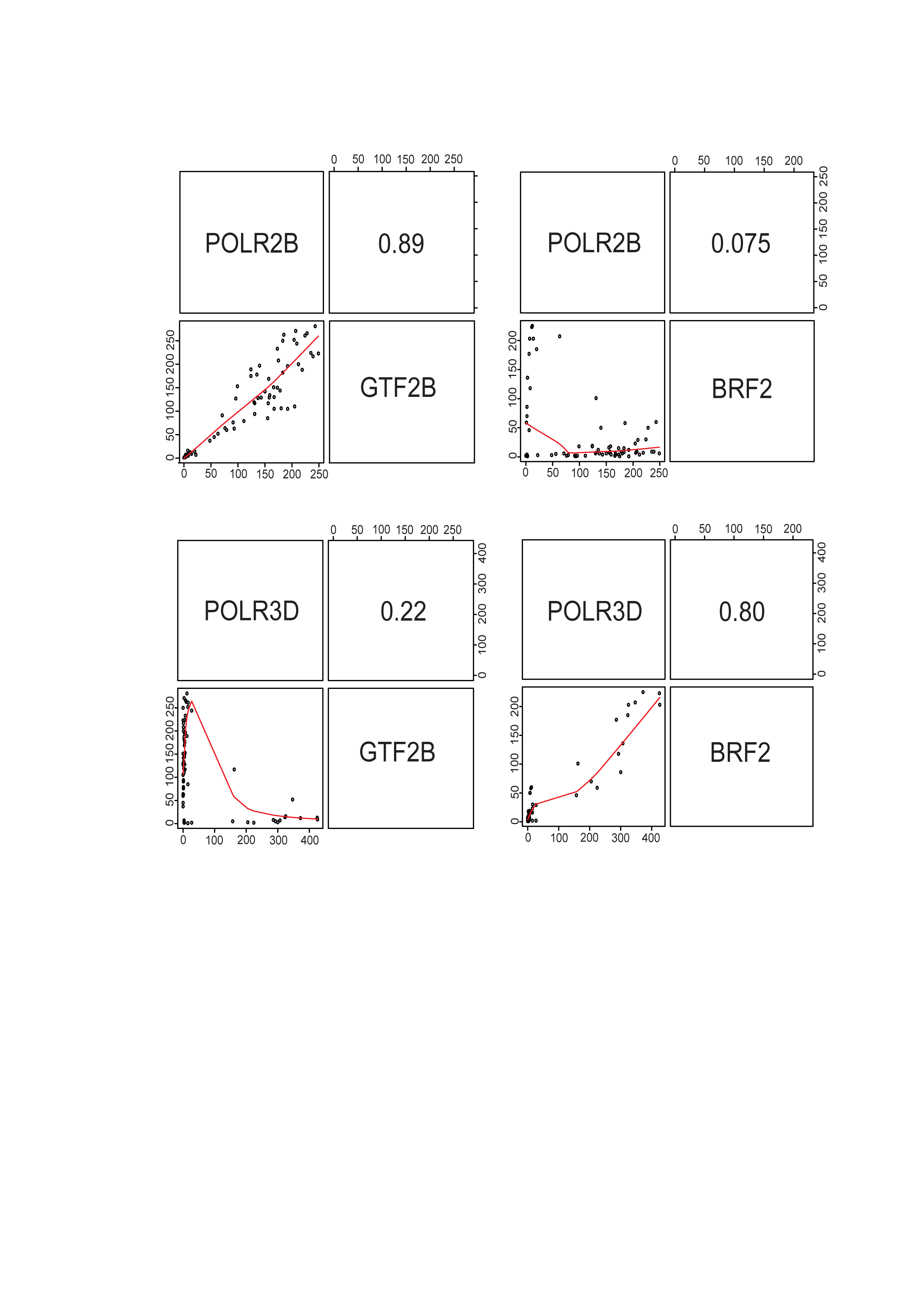

Supplement: Figure S2 — Spearman correlations of scores for genes occupied by pol II, pol III, GTF2B, and BRF2. The scores obtained for the indicated factors refer to all genes listed in Table S1 (except for the RNU2 genes in chr17_random). (TIF) [file pgen.1003028.s002.tif]

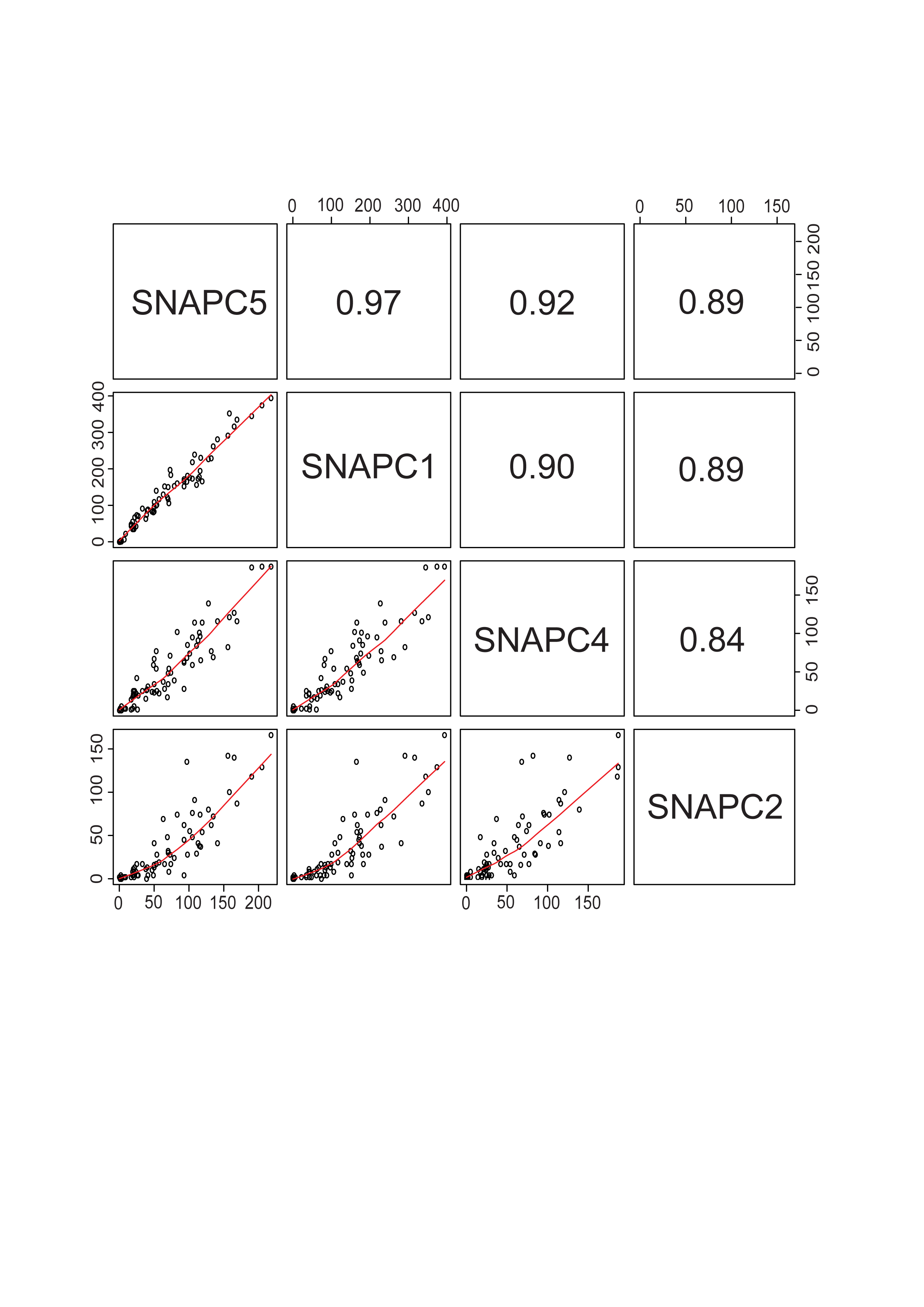

Supplement: Figure S3 — Spearman correlations of scores for genes occupied by all SNAPc subunits tested (SNAPC1, SNAPC2, SNAPC4, SNAPC5). The scores obtained for the indicated factors refer to all genes listed in Table S1 (except for the RNU2 genes in chr17_random). (TIF) [file pgen.1003028.s003.tif]

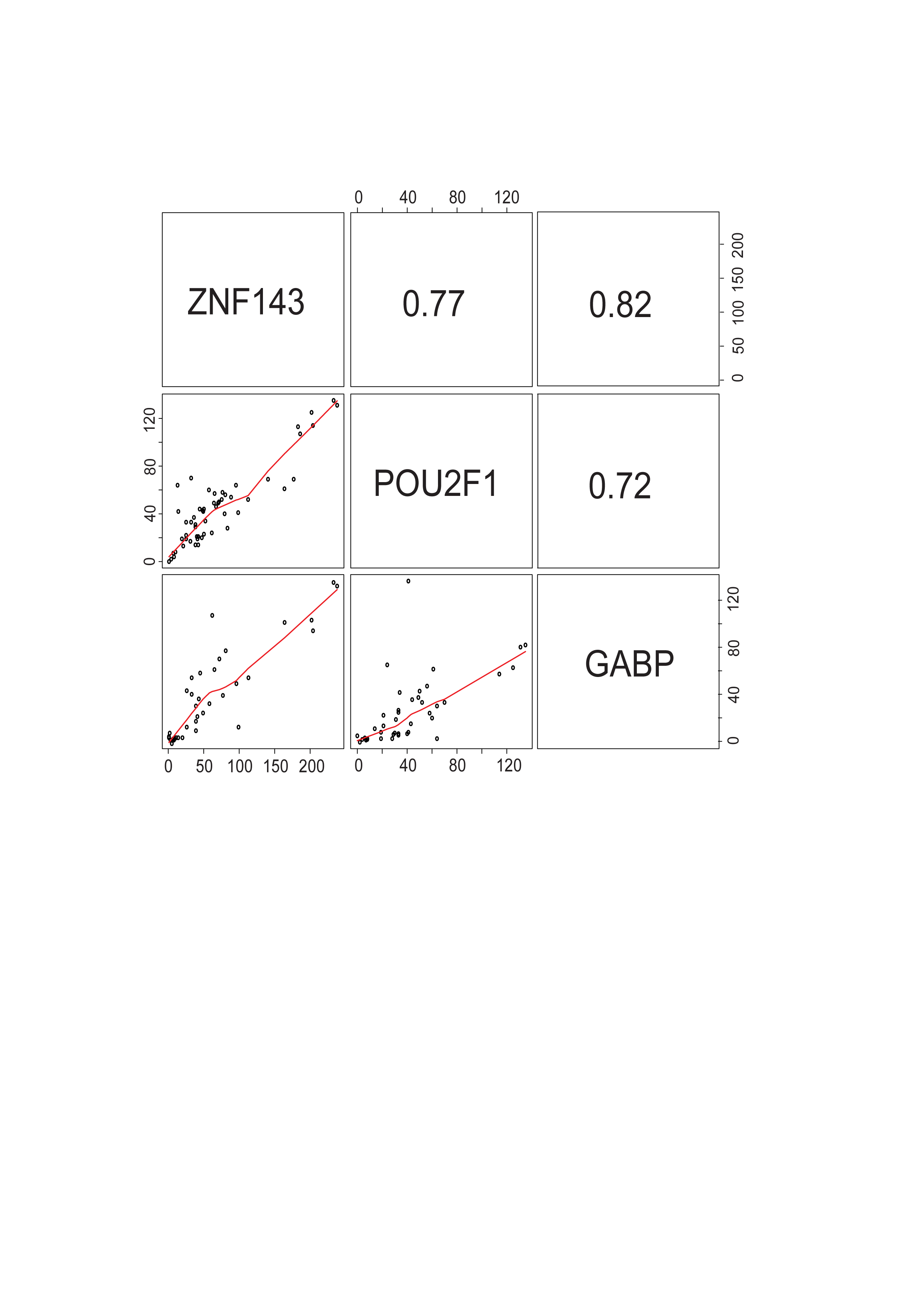

Supplement: Figure S7 — Spearman correlations of scores for genes occupied by ZNF143, POU2F1, and GABP. The scores obtained for the indicated factors refer to all genes listed in Table S1 (except for the RNU2 genes in chr17_random). (TIF) [file pgen.1003028.s007.tif]

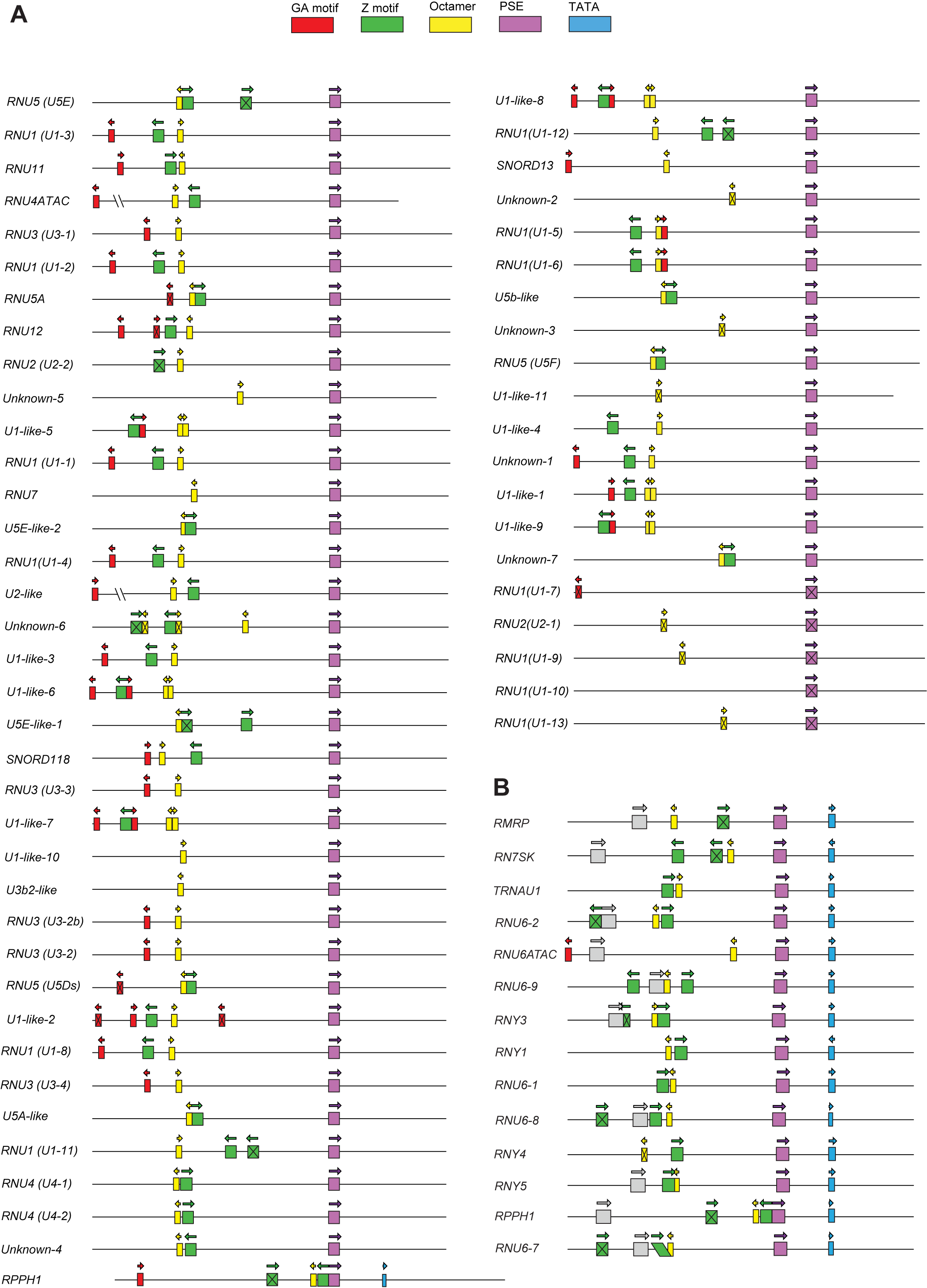

Supplement: Figure S8 — Schematic representation of promoter regions. (A) For each pol II gene in Table S1 (except for the RNU2 genes in chr17_random) as well as the RPPH1 gene, the different motifs found in the promoter region (from −400 to +1 relative to the TSS, except for U2-like, which has a GA motif from −1172 to −1164 upstream of the TSS) are represented by colored boxes as indicated. The direction of the motifs (as shown in the alignments in Figures S9, S10, and S11) is indicated with an arrow. The motifs that appeared not occupied, either because there was no corresponding ChIP-Seq occupancy peak or because they were not closest to the occupancy peak summit, are shown crossed-out (black crosses). In some cases, as for example in the divergent octamers in U1-like-5, two motifs appeared as likely to be occupied. The promoters are aligned relative to the PSEs and ranked by the POLR2B scores. (B) As in (A), but for the pol III genes in Table S1. The grey box indicates a motif (consensus GNC(T/A)G (C/G)(G/C)NN(C/G)(C/T)(C/A)(C/G)(G/C)CG(G/C)(G/A)G) of unknown function found in nearly all type 3 pol III genes. The genes are aligned relative to the TATA box and ranked by the POLR3D scores. (TIF) [file pgen.1003028.s008.tif]
