# Supplementary material for: Genomic Study of RNA Polymerase II and III SNAPc-Bound Promoters Reveals a Gene Transcribed by Both Enzymes and a Broad Use of Common Activators
Source: PLoS Genet. 2012 Nov 15;8(11):e1003028. doi: 10.1371/journal.pgen.1003028 (PMC3499247; doi:10.1371/journal.pgen.1003028)
Supplement: Figure S4 — Alignment of pol III PSEs and TATA boxes. The 5′ flanking sequence of the indicated pol III genes is displayed up to position -1. The PSE and TATA box regions are indicated with a thick line, with the PSE and TATA box as defined in [40] in bold. The numbers refer to the first and last position of the sequences under the thick lines relative to the +1 TSS position. The RNU6 genes are numbered as in [56]. Note that the RPPH1 sequence contains many SNPs. (DOC) [file pgen.1003028.s004.doc]

**PSE**

**TATA**

RMRP TTTTTTTAATCTCACGCCACCAACT -70 TTC**TCACCCTAATCATAAAAC**A -49 CAATTTCTTTAG--GGC-33 **TATAAAATA**CT -23 ACTCTGTGAAGCTGAGGACGTG

RN7SK TGCTGAAGCTCTAGTACGATAAGCA -68 ACT**TGACC-TAAGTGTAAAGT**T -48 GAGACTTCCTT---CAG-33 G**TTTATATA**GC -23 TTGTGCGCCGCTTGGGTACCTC

RNU6 (U6-1) TTATGTTTTAAAATGGACTATCATA -68 TGC**TTACCGTAACTTGAAAGT**A -47 TTTCGATTTCT---TGG-32 C**TTTATATAT**C -22 TTGTGGAAAGGACGAAACACC

RNU6 (U6-2) TATGCTAAATATGAAACCGACCATA -67 AGT**TATCC-TAACCAAAAGAT**G -47 ATTTGATTGA----AGG-33 GC**TTAAAATA**G -23 GTGTGACAGTAACCCTTGAGTC

RNU6 (U6-7) AAGATGGACAGGAAAGCGCTCGATT -68 AGG**TTACCGTAAGGAAAACAA**A -47 TGAGAAACTCCC--GTG-31 CC**TTATAA**GAC -21 CTGGGGACGGACTTATTTGC

RNU6 (U6-8) AAGATGGACAGGAAAGGGCGCGGTT -70 CGG**TCACCGTAAGTAGAATAG**G -49 TGAAAGACTCCC--GTG-33 CC**TTATAA**GGC -23 CTGTGGGTGACTTCTTCTCAAC

RNU6 (U6-9) TTAAATCTCTAGGTCATTTAAGAGA -67 AGT**CGGCC-TATGTGTACAGA**C -47 ATTTGTTCCAG---GGG-32 C**TTTAAATA**GC -22 TGGTGGTGGAACTCAATATTC

RNU6ATAC TGTACCTCCATGGATAGCGAACAAG -67 AAG**TCACCCTCACCGAAAGGC**G -46 AGTGGAGCTTT---CGT-31 CC**TTAAATAAA** -21 GTGCGCAGGGAAGCCGAGGC

RNY1 TAGTCATCAGTAAACTGAAACCAGA -69 ATA**TCACTGTAAGGGGAAAAT**G -48 AACAAATTTGG---GGG-33 C**TTTAAATA**GT -23 TCAAACAGTAGGAGGACTTATT

RNY3 CTTCCTTTTTTTTAGCTCCTGTGAA -70 TAG**TCACCGTAACTATGGTAG**A -49 GATGGAACTTTCGAGGC-31 **TTATATAA**GTA -21 GCAGCGTGCCTTTGTGTTTC

RNY4 GACTTTTTGGAGAATTCTTAAAATA -68 ACT**CATCC-TAACTTATTTAG**A -48 GTAGCCACTTCA--GAG-32 **ATTTATAAAAT** -22 GAAAGTGAAAGCAGTTTTTCT

RNY5 TTACGATCATGGCATAGGCTCTGAA -67 AAG**TCTCCTTACCTAGAAAAG**A -46 CCCTAAGTAG----GCA-32 C**TATAAATAA**C -22 AAGAGACTCACAGGATAACAC

RPPH1 TTTGCATGTCGCTATGTGTTCTGGG -71 AAA**TCACCATAAACGTGAAAT**G -50 TCTTTGGATTTGG-GAA-33 TC**TTATAA**GTT -23 CTGTATGAGACCACTTTTTCCC

TRNAU1 CAACCATCTCACACCTTTCCAAAGG -69 ACG**CGACCATAACTCTAAAAG**G -48 TAAGCTTTTGC---GAT-33 CC**TTATATA**GC -23 TGCGCGGGAATAAGGTTGTCCT
