# Supplementary material for: Genomic Study of RNA Polymerase II and III SNAPc-Bound Promoters Reveals a Gene Transcribed by Both Enzymes and a Broad Use of Common Activators
Source: PLoS Genet. 2012 Nov 15;8(11):e1003028. doi: 10.1371/journal.pgen.1003028 (PMC3499247; doi:10.1371/journal.pgen.1003028)
Supplement: Figure S5 — Alignment of pol II PSEs. The 5′ flanking sequence of the indicated pol II genes is displayed up to position -1. The RPPH1 gene is also displayed. The PSE region is indicated by the thick line with the PSE as defined in [40] in bold. The numbers refer to the first and last position of the sequences under the thick line relative to the +1 TSS position. Note that the following sequences are identical in the region shown: U3-1 and U3-3; U3-2, U3-2b, and U3-4; U1-2, U1-3, and U1-4; U1-5 and U1-6; U1-11 and U1-12. (DOC) [file pgen.1003028.s005.doc]

PSE

_____________

RPPH1|- CTGCAATATTTGCATGTCGCTATGTGTTCTGGG -71 AAA**TCACCATAAACGTGAAAT**G -50 TCTTTGGATTTGGGAATC**TTATAA**GTTCTGTATGAGACCACTTTTTCCC

RNU1|- (U1-1) CAGGGCTGGAAAGGGCTCGGGAGTGCGCGGGGC -64 AAG**TGACCGTGTGTGTAAAGA**G -43 TGAGGCGTATGAGGCTGTGTCGGGGCAGAGGCACAACGTTTC

RNU1|- (U1-2) CAGGGCTGGAAAGGGCTCGGGAGTGCGCGGGGC -64 AAG**TGACCGTGTGTGTAAAGA**G -43 TGAGGCGTATGAGGCTGTGTCGGGGCAGAGCCCGAAGATCTC

RNU1|+ (U1-3) CAGGGCTGGAAAGGGCTCGGGAGTGCGCGGGGC -64 AAG**TGACCGTGTGTGTAAAGA**G -43 TGAGGCGTATGAGGCTGTGTCGGGGCAGAGCCCGAAGATCTC

RNU1|+ (U1-4) CAGGGCTGGAAAGGGCTCGGGAGTGCGCGGGGC -64 AAG**TGACCGTGTGTGTAAAGA**G -43 TGAGGCGTATGAGGCTGTGTCGGGGCAGAGCCCGAAGATCTC

RNU1|- (U1-5) TCAGGGCTAGGAAGGCTCGGGGGTGCGCGGGGC -64 AAG**TGACCATGTGTGTAAAGG**G -43 TGAGGTATATGGAGCTGTGACAGGGCAGAAGTGTGTGAAGTC

RNU1|- (U1-6) TCAGGGCTAGGAAGGCTCGGGGGTGCGCGGGGC -64 AAG**TGACCATGTGTGTAAAGG**G -43 TGAGGTATATGGAGCTGTGACAGGGCAGAAGTGTGTGAAGTC

RNU1|- (U1-7) GGGGGTGGGGGGCGGTGGGGGGTTGCGCGTGGG -64 AAG**TGACCGTGCGTGTAAGGG**G -43 TGAGGCGTATGGAGCTGTGGCAGGGCGGAGGCGTATGATCTC

RNU1|- (U1-8) GGGGGGGGGGGGGGGGGGGGGGCTGCGCGGGGC -64 AAG**TGACCGTGTGTGTAAAGG**G -43 TGAAGCGTGTGAGGCTGTGGCGGGGCGGAGGTGCAAGAGCTC

RNU1|- (U1-9) TTTTATTTTTCTGCATATGACTAGCTGATTATC -69 TCA**GCACCATTTTTTGGATCT**G -48 TGTATGAATCTACAATTATCTTAACTCTTGATTAAAAAAGAAAACTC

RNU1|+ (U1-10) TAAAAGTTACTCTAATCTTTGAAATAGAAAAAT -62 ATC**TTACTTTTATGATCAATG**T -41 TTTGTTACAAACTGTCTCAATTATAGAAACAGGCTTTTGC

RNU1|+ (U1-11) GGAGAGGGTGAGGGGGCTGGGGCCGCACGAGAG -64 AAG**TGACCGTGTGTTGAGAGT**G -43 TGGTGGGCGCGAGGGTATGAGAGGAAGCCGCACGGCCAGTCC

RNU1|- (U1-12) GGAGAGGGTGAGGGGGCTGGGGCCGCACGAGAG -64 AAG**TGACCGTGTGTTGAGAGT**G -43 TGGTGGGCGCGAGGGTATGAGAGGAAGCCGCACGGCCAGTCC

RNU1|+ (U1-13) GCTGGGGAGGTTTTTGTCAGGGATAAGGATGGG -67 GGC**AAACTATGTTAAGGGAAG**T -46 GATGACAAGAAAGCCTTCCCTGCGATTAAGAAATTATAATAATAT

U1-like-1|- AGGGCTAGGATGGCTCCTGGATGCGCGTGACGC -70 AAG**TGACCTTGCGTGTAAAGG**G -49 TGAGGCATATGAGGCTGCGGCGGGGCGGAGGGGCGTGAGCTTATACTT

U1-like-2|+ GGCATGGGGGCGGGGTGGGGGAATGCGCGGGGC -64 AAG**TGACCGTGCGTGTAAGGG**G -43 TGAGGCGTATGGAGCTGTGGCAGGGCGGAGGTGCGTTCATTC

U1-like-3|+ GAGGGCTGGGGGGAGGGGGGGTGTGCGCGGGGC -67 AAG**TGACCGTGCGTGTAAAGG**G -46 TGAAGCGTGTGAGGCTGCCGGCGGGGCGGAGAGTGCAATAACTC

U1-like-4|- TCAGGGCTAGGAGGCCTCGAGGGTGCGCGGGGC -64 AAG**TGACCGTGCGTGTAAAGG**G -43 TGAGGCGTATGAGGCTGTGCCGGGGCGGAAGCGTGCAGACTC

U1-like-5|+ GAGGGCTGGGGGGGGCGGGGAGGTGCGCGGGGC -64 AAG**TGACCGTGCGTGTAAAGG**G -43 TGAAGCGTGTGAGGCTGTGGCGGGGCGGAGGTGCAAAAGCTC

U1-like-6|+ GGGTGGGGTGGGGGGGGGGGGCGTTCGCGGGGC -64 AAG**TGACCGTGCGTGTAAAGG**G -43 TGAAGCGTGTGAGGCTGTGGCGGGGCGGAGGTGCAAAAGCTC

U1-like-7|- GGGGGGGGTGGGGGGGGGGGGCGTGCGCGGGGC -64 AAG**TGACCGTGCGTGTAAAGG**G -43 TGAAGCGTGTGAGGCTGTGGCGGGGCGGAGGTGCAAAAGCTC

U1-like-8|- GCGGGGGGGGTGGGGGGGGGGCGTGCGCGGGGC -64 AAG**TGACCGTGCGTGTAAAGG**G -43 TGAAGCGTGTGAGGCTGTGGCGGGGCGGAGGTGCAAAAGCTC

U1-like-9|+ AGGGCTAGGATGGCTCCTGGATGCGCGTGACGC -70 AAG**TGACCTTGCGTGTAAAGG**G -49 TGAGGCATATGAGGCTGCGGCGGGGCGGAGGGGCGTGAGCTTATACTT

U1-like-10|- TTCGAAGCGATGCCTAAAAACAACTCATAATAC -73 AAT**TTGCCATGAGTATGTTGT**G -52 TGTTGAGGACGATGGTAAGGTATTAGCAATATTAAGTAAATAATGTCTTTG

U1-like-11|- AGAAATTAGGGGGTCTAAAGAGCACATGAGAAA -63 AAT**GATCCTATATTTAGAGTG**G -42 TTTGAATCTAAGGGTGAACTCTCAGGCAGCGCTGGGGACTC

RNU2|-(U-1) GCGCCATTGTATTCCAGCCTGGGCACCAAGAGC -68 GAA**ACTCCATCTTAAAAAAAA**A -47 ATAAAAATAAAAAAATTTTAAAAAGAAAAAAAGAAAAAGAATTAAA

RNU2|-(U2-2) TATTGGGAGATAATTTAACATTTAGTGCCTGGA -63 TAG**TTACCATAACTGGTTGGA**A -42 GATGGGAAGGATAAGGCCGCCGAGGCGACCGAAGTAAAGGT

U2-like|- GGAAGAGAGTGCTTCTTTACTTGAGGATCATTT -66 TTC**TTACCGTGACCTCAGGAT**G -45 AGTGGGAGAAGGAGTGCTGTGGCCGAAAGAAAGGGCTTGTTTCT

RNU3|+ (U3-1) TTGTTTCCTAATGGCATCGGAACTAGCGAAAGT -65 TTC**TCGCCATCAGTTAAAAGT**T -44 TGCGGCAGATGTAGACCTAGCAGAGGTGTGCAAGGAGGCCGTT

RNU3|+ (U3-2) TTGTTTCCTAATGGCATCGGAACTAGCGAAAGT -65 TTC**TCGCCATCAGTTAAAAGT**T -44 TGCGGCAGATGTAGACCTAGCAGAGGTGTGCGAGGAGGCCATT

RNU3|- (U3-2b) TTGTTTCCTAATGGCATCGGAACTAGCGAAAGT -65 TTC**TCGCCATCAGTTAAAAGT**T -44 TGCGGCAGATGTAGACCTAGCAGAGGTGTGCGAGGAGGCCATT

RNU3|- (U3-3) TTGTTTCCTAATGGCATCGGAACTAGCGAAAGT -65 TTC**TCGCCATCAGTTAAAAGT**T -44 TGCGGCAGATGTAGACCTAGCAGAGGTGTGCAAGGAGGCCGTT

RNU3|- (U3-4) TTGTTTCCTAATGGCATCGGAACTAGCGAAAGT -65 TTC**TCGCCATCAGTTAAAAGT**T -44 TGCGGCAGATGTAGACCTAGCAGAGGTGTGCGAGGAGGCCGTT

U3b2-like|- TAATATTTTCGAGAGGAAAATGTGCTCACGAAA -64 TAC**TCACCATCAGCATAATAT**G -43 TGTGAGTTTCTTTCGCATGTGGAAGGCACATAAATGGACATT

RNU4|- (U4-1) GGAACACGTCGTATACACGGACACACGGGCAGG -65 CAC**TCACCCTCAATGTAATGG**T -44 AGTCATCATCCGTGGGGGAGCGGGGCGCGAACAGAACCTTTCC

RNU4|- (U4-2) AAGCAATAAGTGAAGATTTTTCCATAGGCCCTA -64 AAC**TCACCTTTGCGAAATAGG**A -43 AGCTGGTTTATTGGGAGTGATGAGCAGGGGGCGTAACAAATT

U4ATAC|+ GCTACCAGACCGACTAGGGCGAGGCTCACGAAT -63 TAA**TTACCACAACCCTACCAG**G -42 TATTGGCGCTTCCTGCTTGCAGCCCAGGGACTTTCTATTAT

RNU5|+ (U5A) TAAACTTTCTCAGGTAGTAACCCTTGGGATTAG -63 TAG**ACACCATCAGTGTACTAG**G -42 AATTGCAGTTACCCGAAAATTGAGTTACAGAAGTAACTGGT

RNU5|- (U5Ds) GAGACAAAAACAGAAGTCACTCTTTTGGGTTAA -65 TAG**TAACCATTGCTAATCTAG**T -44 AGTGACCGTCCCCCGAGGACTGTGTGCAACCATTCCAACACAT

RNU5|+ (U5E) GGCTGAAGTCAATAGCTCTTTTGGGCCGAAGGA -66 AAG**TTACCATTACCCGTTTAG**G -45 AGTAGCCGTTACCTGAGAACTGTAGTGTCGACGACTGATGTTAT

RNU5|- (U5F) ACAGTGGAAGAAAAGCTTCTGTCTGCAGGTCCA -65 AAG**GCACCGTAAGTAGAGGGA**G -44 ACCAGTCAATAGCTGGGAAGCCAGGCAAAAGGCTAACAGGCAG

U5A-like|- AACTGTAGGCAGTAATTCTTTTTGTGCGAACTG -65 TAG**GCACCATCGGCGTACTAG**G -44 AGTTGCGGTTACTGCAACAATGAGTTGAACTAATTTGTAGCAT

U5b-like|+ ATTTTTTTCTAGGTATAGAACCTTGGCATTCAC -63 TAG**TCACCATCACTATACTAG**G -42 AGTTTCTGTTACCCGAGAAACGAGTTATGAAATTAACAAGC

U5E-like-1|- GGCAGAAGTCAATAGCTCTTTTGGGCAGAAAGA -66 AAT**TTACCATTAGCCTGTTGG**G -45 AGTAGCCATTACCTGAGAACTGAGTGCCACTCATCGAAGTTCTT

U5E-like-2|- CTACATATAGGGAGTGCGTACCACGCAGGCATC -65 CAA**CAACCATAAGTGTGTTAA**G -44 TGTTAGTTCTCCCTGCGAGGTTCGAAGCGGAAGTCACGAATAT

RNU7|+ ATACAGTAATAGGAACAAGAAAAAAGTCACCTA -62 AGC**TCACCCTCATCAATTGTG**G -41 AGTTCCTTTATATCCCATCTTCTCTCCAAACACATACGCA

SNORD118|- (U8) AAATAATAGGATTGCTCATACCAGCGCGTTATG -63 AAC**TCACCCTAGCTTGTAACG**G -42 AATCTTTTTCACTGAGTGCAGAATGTCGGCTGTTTGTCTGT

RNU11|+ (U11) AGGAAGTTGAAATAAAGAGTTGCTTGGATTTTG -64 TGT**TCACCTTTACCAAAAAAT**G -43 GATTTGGTAACACTGCCACCCTGCTTTGGTGACAGAGAAAGC

RNU12|+ (U12) GAACTGGATCATGACCTCAACAGTCAACTACGA -63 TAG**TCATCATACGCCTAATGA**G -42 AATAGAATTCATTACCTAGGAAATAAACTAAAAACGTCCTT

SNORD13|+ (U13) TTATACGTGAAGAATGGATGTATCGCATTACGG -63 TAG**TCACCCTATGTGTAAATT**A -42 GTGGCACATACTTGGCACTCCTTAATGTCAACTATAAGATG

UNKNOWN-1|+ TGATTGGGGATTGGCTGGGGTGGTGCTCGGGGC -67 AAG**TGACCGTGCGTGTAAAGG**G -46 TGAGGCGTATGGAGCTGTGGCGGGGCGGAAGTTCACATGTGTGGT

UNKNOWN-2|- AAACAGCCCGTTTGTTTTATCTCCTAATTAGCA -65 TAC**TCACCCTCAGCTGAAAAT**G -44 AGTCCATTAAAGGAGACTCTGCTCCAGGCTCAGCTCCTCCTTT

UNKNOWN-3|- TCCAAAGTACAGTGTAAGAACAGACTGGAAGAG -65 AAG**TTACCCCGATGACTTGGT**T -44 TGGAAGGGGTTAAGGCACCAGTCATCCTCTTCTAAAGTGATTT

UNKNOWN-4|+ AAAAATCCCTCCACAACAACAACAACAAAAAAG -63 AAC**TCACCATAACTAAGAGAA**G -42 AGTTGGGCATCATGGGAAAACTCAGGAAGAGCCTGCGGGAG

UNKNOWN-5|+ AGAGCTGAGAATTCACTTGAAAACGTAGCCAAC -60 TAT**AAACCATGCCCAAAGGCT**T -39 GCTGTTTTCAACAGGCCTATTGAGAAACGAAGTTGTCA

UNKNOWN-6|- CAACGTAGGAGAGAGAGTTATTCAGGGTGCTAT -62 CTG**TCACCCTGAGGTTAGGAT**G -41 TGTGGGAGAAGGAGTACAGTGGCCGATCGTTAACATTTTC

UNKNOWN-7|- AATATATTTACAAGTTAAAAGCCGTAGGTGTCC -63 TTT**AAACCTGCTGTTTTGAAG**A -42 ATGATACTTTTCCTTACACATTCGCCTGAAAGAAGTGCTGT
