# Supplementary material for: Genomic Study of RNA Polymerase II and III SNAPc-Bound Promoters Reveals a Gene Transcribed by Both Enzymes and a Broad Use of Common Activators
Source: PLoS Genet. 2012 Nov 15;8(11):e1003028. doi: 10.1371/journal.pgen.1003028 (PMC3499247; doi:10.1371/journal.pgen.1003028)
Supplement: Figure S6 — Alignment of 3′ boxes. Sequences resembling 3′ boxes (consensus sequence GTTT N1–4 AANAA/G N AGA, see [40]) within the 100 nt following the RNA-coding sequence (+1 to +100, with the 3′ end of the RNA coding region set at 1) were identified manually. These motifs were used to generate a matrix with GLAM2 [20] (which allows gaps), which was then used to search for motifs in all sequences with GLAM2SCAN [20]. The GLAM2SCAN analysis confirmed all motifs except the two shown in italics, and identified motifs in the novel un-annotated genes as well as some additional motifs (underlined). In the RPPH1 gene, the best match to a 3′ box was found inside the RNA coding sequence. (DOC) [file pgen.1003028.s006.doc]

**3’ box**

**____________________**

RPPH1 -73 GTT C--- AATGG C TGA -61

RNU1(U1-1) +11 GTT TC-- AAAAA C AGA +24

RNU1(U1-2) +11 GTT TC-- AAAAA C AGA +24

RNU1(U1-3) +11 GTT TC-- AAAAA C AGA +24

RNU1(U1-4) +11 GTT TCT- AAAAG T AGA +25

RNU1(U1-5) +11 ATG A--- AAAAA T AGA +23

RNU1(U1-6) +11 ATG A--- AAAAA T AGA +23

RNU1(U1-7) +10 CTG GA-- AAACG C AGA +24

RNU1(U1-8) +11 GTT TA-- AAGAA T AGT +24

RNU1(U1-9)

RNU1(U1-10) +89 GTT T--- GATGT T AGA +101

RNU1(U1-11) +12 GTT G--- AAAGG T AGC +24

RNU1(U1-12) +12 GTT G--- AAAGG T AGC +24

RNU1(U1-13)

U1-like-1 +11 GTT TT-- AAAAG T GGA +24

U1-like-2 +10 GTG TA-- AAAAG C AGT +23

U1-like-3 +11 GTT TT-- AAAAA T AGG +24

U1-like-4 +23 GTG G--- AAAGA T AGA +35

U1-like-5 +12 GTG CG-- AATAG T AGG +25

U1-like-6 +11 GTG CG-- AATAG T AGG +24

U1-like-7 +12 GTG CG-- AATAG T AGG +25

U1-like-8 +12 GTG CG-- AATAG T AGG +25

U1-like-9 +11 GTT TT-- AAAAG T GGA +24

U1-like-10 +19 GTT T--- AAAAG A CGG +31

U1-like-11 +10 GTT T--- ATAAA A GGC +23

RNU2(U2-1) +32 ATT A--- AAAGA A ATA +44

RNU2(U2-2) +26 GTT T--- AATGG A AGA +38

U2-like +11 TTT ACA- AAGAA C AGA +23

RNU3(U3-1) +16 GCT C--- AATGA C AGA +28

RNU3(U3-2) +16 GCT C--- AATGA C AGA +28

RNU3(U3-2b) +16 GCT C--- AATGA C AGA +28

RNU3 (U3-3) +16 GCT C--- AATGA C AGA +28

RNU3 (U3-4) +16 GCT C--- AATGA C AGA +28

U3b2-like -2 *TCT AGA- GAAGG C AGT* +12

+44 ATG TT-- AATAG T AGT +57

RNU4 (U4-1) +12 GTC TA-- AAGAA A AGG +25

RNU4 (U4-2) +13 GTT G--- AACAA C AGA +25

RNU4ATAC +27 GTT CA-- AACAG C AGT +40

RNU5 (U5A) +10 ATC C--- AACAA T AGA +22

RNU5 (U5Ds) +25 GTT TA-- AAAAT C AGA +38

RNU5 (U5E) +10 GTT TT-- AAAAA C AGA +23

RNU5 (U5F) +10 GTT ACT- AAAGA G AGA +24

U5A-like +8 GTT TTAT AAAAA A AGA +23

U5B-like +10 GTT AA-- AAAAT C AGA +23

U5E-like-1 +9 GTT TT-- AAAAG T AGA +22

U5E-like-2 +29 ATT A--- AAAGT T AGG +41

RNU7 (U7) +13 GTT GCC- AATGA T AGA +27

SNORD118 (U8) +31 GCT G--- ATTAG C AGA +43

RNU11 (U11) +15 *GTT AGG- CGAAA T ATT* +29

+56 GTA ACT- GAAAA G AGA +70

RNU12 (U12) +12 GCC TA-- AAAAG T AGA +26

SNORD13 (U13) +5 CTT C--- AAGGA T CGA +17

UNKNOWN-1 +15 GCT GC-- AAGGT C AGG +28

UNKNOWN-2 +160GTT C--- AAGAG C AGT+172

UNKNOWN-3 -77 GTG CT-- AAAAT G GGA -64

+76 AAG A--- AAAAG A AGA +88

UNKNOWN-4 +37 GAC C--- AAAGG C AGG +49

UNKNOWN-5 +70 CTT C--- AAACA A AGG +82

UNKNOWN-6 -29 GTC GCT- AACGG G AGA -15

UNKNOWN-7 -62 TTT TTAG ATTAA T AGA -47
