# Supplementary material for: Genomic Study of RNA Polymerase II and III SNAPc-Bound Promoters Reveals a Gene Transcribed by Both Enzymes and a Broad Use of Common Activators
Source: PLoS Genet. 2012 Nov 15;8(11):e1003028. doi: 10.1371/journal.pgen.1003028 (PMC3499247; doi:10.1371/journal.pgen.1003028)
Supplement: Figure S9 — Alignment of octamer sequences. Sequences similar to the POU2F1 binding site (octamer) located within peaks of POU2F1 occupancy in the 5′ flanking regions of the indicated genes, except for the octamers in RNY4, U1-9, U1-13, and unknown-3, which are not occupied. In unknown-6, the second octamer closer to the TSS is the best centered under the peak summit even though it is a less good octamer than the one further upstream. The numbers refer to the first and last position of the sequences shown relative to the +1 TSS position. The genes not shown in the list (U1-7, U1-10, and U1-13) have no matches (with up to two mismatches) to the octamer up to 400 bp upstream of the TSS. All octamer sequences present in the alignment are shown as boxes in Figure S8A and S8B. Sequences labeled with an asterisk are the closest to the POU2F1 peak summit on the corresponding promoter, and were used to generate the octamer LOGO shown in Figure 4C. The U1-like-1/-5/-6/-7/-8/-9 promoter regions have two overlapping octamers of similar quality located in each case near the POU2F1 peak summit; one or both of these motifs may be occupied. (PDF) [file pgen.1003028.s009.pdf]

Octamer

RMRP|-	-210	ATGCAAAT	-217*	-
RN7SK|+	-126	TAGCAAAT	-134*	-
RNU6|- (U6-1)	-214	ATGCAAAT	-221*	-
RNU6|+ (U6-2)	-234	ATGCAAAA	-241*	-
RNU6|+ (U6-7)	-214	ATGCAAAT	-221*	-
RNU6|- (U6-8)	-215	ATGCAAAT	-222*	-
RNU6|+ (U6-9)	-217	ATGCAAAT	-224*	-
RNU6ATAC|-	-123	CCACAAAT	-130*	-
RNY1|-	-220	ATGCAAAA	-227*	-
RNY3|+	-242	ATGCAAAT	-235*	+
RNY4|+	-255	TTGCAAAT	-262	-
RNY5|+	-209	TTACAAAT	-216*	-
RPPH1|-	-90	ATGCAAAT	-97*	-
TRNAU1|-	-211	ATGTAAAT	-204*	+

RNU1|- (U1-1)	-220	ATGTAGAT	-213*	+
RNU1|- (U1-2)	-219	ATGTAGAT	-212*	+
RNU1|+ (U1-3)	-219	ATGTAGAT	-212*	+
RNU1|+ (U1-4)	-219	ATGTAGAT	-212*	+
RNU1|- (U1-5)	-218	ATGTAGAT	-211*	+
RNU1|- (U1-6)	-218	ATGTAGAT	-211*	+
RNU1|- (U1-8)	-235	ATGTAGAT	-228*	+
RNU1|- (U1-9)	-178	ATGAAAAT	-185	-
RNU1|+ (U1-11)	-222	ATGCAGAT	-215*	+
RNU1|- (U1-12)	-222	ATGCAGAT	-215*	+
RNU1|+ (U1-13)	-122	ATGTAAAT	-115	+
U1-like-1|-	-225	ATAGAAAT	-232*	-
	-226	ATGTAGAT	-219*	+
U1-like-2|+	-226	ATGCAGAG	-219*	+
U1-like-3|+	-230	ATGTAGAT	-223*	+
U1-like-4|-	-218	ATGTAGAT	-211*	+
U1-like-5|+	-212	ATAGAAAT	-219*	-
	-213	ATGTAGAT	-206*	+
U1-like-6|+	-232	ATAGAAAT	-239*	-
	-233	ATGTAGAT	-226*	+
U1-like-7|-	-226	ATGTAGAT	-219*	+
	-225	ATAGAAAT	-232*	-
U1-like-8|-	-223	ATAGAAAT	-230*	-
	-224	ATGTAGAT	-217*	+
U1-like-9|+	-225	ATAGAAAT	-232*	-
	-226	ATGTAGAT	-219*	+
U1-like-10|-	-220	ATGTAGAT	-213*	+
U1-like-11|-	-221	ATGTAGAT	-214	+
RNU2|- (U2-1)	-213	ATGCAAAA	-206	+
RNU2|- (U2-2)	-223	ATGCAAAA	-216*	+
U2-like|-	-233	ATGCAAAT	-226*	+
RNU3|+ (U3-1)	-226	ATGCTAAT	-219*	+

RNU3|+ (U3-2)	-226	ATGCTAAT	-219*	+
RNU3|- (U3-2b)	-226	ATGCTAAT	-219*	+
RNU3|- (U3-3)	-226	ATGCTAAT	-219*	+
RNU3|- (U3-4)	-226	ATGCTAAT	-219*	+
U3b2-like|-	-218	ATGCAAAT	-225*	-
RNU4|- (U4-1)	-222	ATGCAAAT	-229*	-
RNU4|- (U4-2)	-220	ATGCTAAT	-227*	-
RNU4ATAC|+	-228	ATGCAAAT	-221*	+
RNU5|+ (U5A)	-196	ATGCAAAT	-203*	-
RNU5|- (U5Ds)	-209	ATGTAAAT	-216*	-
RNU5|+ (U5E)	-215	ATGCAAAT	-222*	-
RNU5|- (U5F)	-221	ATGCAAAT	-228*	-
U5A-like|-	-203	ATGCAAAT	-210*	-
U5b-like|+	-205	ATGCAAAT	-212*	-
U5E-like-1|-	-217	ATGCAAAT	-224*	-
U5E-like-2|-	-211	ATGCAAAT	-218*	-
RNU7-1|+ (U7)	-198	ATGCAAAT	-205*	-
SNORD118|- (U8)	-249	ATGTAAAT	-242*	+
RNU11|+ (U11)	-212	ATGCAAAT	-219*	-
RNU12|+ (U12)	-198	ATGCAGAT	-205*	-
SNORD13|+ (U13)	-201	ATGCAAAT	-208*	-
UNKNOWN-1|+	-230	ATGTAGAT	-223*	+
UNKNOWN-2|-	-102	ATGCAAAT	-109	-
UNKNOWN-3|-	-124	ATGCAGAA	-117	+
UNKNOWN-4|+	-219	ATGCAAAT	-226*	-
UNKNOWN-5|+	-133	ATGCAAAT	-126*	+
UNKNOWN-6|-	-264	CTGTAAAT	-271	-
	-220	ATGCAAAT	-213	+
	-111	ATCCAAAT	-118*	-
UNKNOWN-7|-	-117	ATGCAAAT	-124*	-
