# Supplementary material for: Genomic Study of RNA Polymerase II and III SNAPc-Bound Promoters Reveals a Gene Transcribed by Both Enzymes and a Broad Use of Common Activators
Source: PLoS Genet. 2012 Nov 15;8(11):e1003028. doi: 10.1371/journal.pgen.1003028 (PMC3499247; doi:10.1371/journal.pgen.1003028)
Supplement: Figure S10 — Alignment of Z-motifs. Sequences similar to ZNF143 binding sites (Z-motif) located within peaks of ZNF143 occupancy in the 5′ flanking regions of the indicated genes, except for the Z-motif in the RMRP gene and the more upstream Z-motif in the RPPH1 gene, which are not under a ZNF143 peak. The numbers refer to the first and last position of the sequences shown relative to the +1 TSS position. The sequences in bold were identified by a MAST [20] search with the consensus Z-motif [25] or a similar motif identified by a MEME [20] de novo search of motifs present under ZNF143 peaks. All Z-motifs present in the alignment are shown as boxes in Figure S8A and S8B. Sequences labeled with an asterisk are the closest to the ZNF143 peak summit on the corresponding promoter, and were used to generate the ZNF143 binding site LOGO shown in Figure 4C. (PDF) [file pgen.1003028.s010.pdf]

		**Z-motif**	
RMRP|-	-149	**TAGCCACAATGCCTCTG**	-133
RN7SK|+	-198	**TATCCAGAATGCCTTGC**	-214*
	-142	**TTCCCAAAATGCTAAAG**	-158
RNU6|- (U6-1)	-239	**TTCCCATGATTCCTTCA**	-223*
RNU6|+ (U6-2)	-316	**ATCCCAGCGTTCTCTGC**	-332
	-228	**CACCCACAATCCACCGC**	-212*
RNU6|+ (U6-7)	-323	**CTCCCGGCATGCTCTGC**	-307
	-244	**TACCCAGGGTGCCCCGG**	-228*
RNU6|- (U6-8)	-323	**CTCCCGGCATGCTCCGC**	-307
	-245	**TACCCAGGGTGCCCCGG**	-229*
RNU6|+ (U6-9)	-262	**CTCCCAGAAGTCTTTGG**	-278*
	-199	**TTCCCAGCAGCCACCGT**	-183*
RNY1|-	-213	**TTCCCAGGATGCTTAGC**	-197*
RNY3|+	-275	**CTCCCACGGTGCCCCCG**	-291
	-234	**CACCCACAATGCTTTGA**	-218*
RNY4|+	-218	**TTCCCATCATGCAACTA**	-202*
RNY5|+	-228	**TACCCACAATGCATTTG**	-212*
RPPH1|-	-163	**CACCCAGCGCGCGTGCG**	-147
	-70	TTCCCAGAACACATAGC	-86*
TRNAU1|-	-231	**TTCCCAGAATGCGTGGC**	-215*
RNU1|- (U1-1)	-244	CACCGACAGTGCCTTGC	-260*
RNU1|- (U1-2)	-243	CACCGACAGTGCCTTGC	-259*
RNU1|+ (U1-3)	-243	CACCGACAGTGCCTTGC	-259*
RNU1|+ (U1-4)	-243	CACCGACAGTGCCTTGC	-259*
RNU1|- (U1-5)	-242	CACCGACAGCGCCTTCC	-258*
RNU1|- (U1-6)	-242	CACCGACAGCGCCTTCC	-258*
RNU1|- (U1-8)	-259	CACCGACAGTGCCTTTC	-275*
RNU1|+ (U1-11)	-133	CACCCCGACAGCACTCG	-149*
	-100	**CCCCCTCGCTCCACCGT**	-116
RNU1|- (U1-12)	-133	CACCCCGACAGCACTCG	-149*
	-100	**CCCCCTCGCTCCACCGT**	-116
U1-like-1|-	-250	CACCGACAACGCCTTCT	-266*
U1-like-2|+	-250	CACCGACAGCGCCTTTC	-266*
U1-like-3|+	-254	CACCGACAGTGCCTTTC	-270*
U1-like-4|-	-279	ATCCCACCCTCCGCCAC	-295*
U1-like-5|+	-275	**GCCCCACCCTCCGCCGA**	-291*
U1-like-6|+	-295	**GCCCCACCCTCCGCCGA**	-311*
U1-like-7|-	-288	**GCCCCACCCTCCGCCGA**	-304*
U1-like-8|-	-286	**GCCCCACCCTCCGCCGA**	-302*
U1-like-9|+	-287	GCCTCACCCAACGCCCC	-303*
RNU2|- (U2-2)	-259	**ATCCCAGGATCCTAAAA**	-243
U2-like|-	-192	TTCCCATAGCTCCTGAG	-208*
RNU4|- (U4-1)	-217	**TTCCCCAGCGTCCCAAG**	-201*
RNU4|- (U4-2)	-215	**CTCCCATAGTGCTTTGC**	-199*
U4ATAC|+	-189	**TACCCAGAAGACCGCGC**	-205*
RNU5|+ (U5A)	-196	TACCCATAATACACTGC	-180*
RNU5|- (U5Ds)	-209	**TACCCACAATGCACTGC**	-193*
RNU5|+ (U5E)	-215	**TACCCACAATGCATTGC**	-199*
	-123	**GTCCCAGAATTGTCTGA**	-107

RNU5|- (U5F)	-222	**ATCCCAGAATGCATTGT**	-206*
U5A-like|-	-203	TACCCATAGTACCCTGC	-187*
U5b-like|+	-205	**TACCCATAATGCACTGC**	-189*
U5E-like-1|-	-217	**TACCCACAATGCATTGT**	-201
	-123	**GTTCCAGAATTGTCTGA**	-107*
U5E-like-2|-	-211	**TACCCATGGTGCACCAC**	-195*
SNORD118|- (U8)	-186	**TTCCCAGAATGCTCCGA**	-202*
RNU11|+ (U11)	-240	**TTCCCAGCAAGCCCTGA**	-224*
RNU12|+ (U12)	-237	TTCCCCGTGAGCCTTCG	-221*
UNKNOWN-1|+	-251	CACCAACAGTGCCTTTC	-267*
UNKNOWN-4|+	-196	**TTCCCAGGAGACTCCTG**	-212*
UNKNOWN-6|-	-288	**TTCCCAAGATTCCAGTG**	-272
	-221	**GCCCCGCCGTGCCCTGC**	-237*
UNKNOWN-7|-	-117	**TAACCATGGTGCACTGC**	-101*
