# Supplementary material for: Genomic Study of RNA Polymerase II and III SNAPc-Bound Promoters Reveals a Gene Transcribed by Both Enzymes and a Broad Use of Common Activators
Source: PLoS Genet. 2012 Nov 15;8(11):e1003028. doi: 10.1371/journal.pgen.1003028 (PMC3499247; doi:10.1371/journal.pgen.1003028)
Supplement: Figure S11 — Alignment of GA-motifs. Sequences similar to GABP binding sites (GA-motif) present within 400 bp upstream of the TSSs of the genes in Table S1 (except for the RNU2 genes in chr17_random) identified by MAST [20] with the GABP consensus sequence [25] are indicated in bold, a few additional ones found manually (located under GABP peaks of occupancy) are indicated in standard font. All GA-motifs present in the alignment are shown as boxes in Figure S8A and S8B. The sequences located within peaks of GABP occupancy and closest to the peak summit are indicated with an asterisk and were used to generate the GABP binding site LOGO in Figure 4C. The numbers refer to the first and last position of the sequences shown relative to the +1 TSS position. For some U1-like genes, two GABP sites of similar quality were identified under the GABP peak. (PDF) [file pgen.1003028.s011.pdf]

GA-motif

RNU6ATAC|-	-362	**ACGGAAGTG**	-370*
RNU1|- (U1-1)	-319	**GAGGAAGTG**	-327*
RNU1|- (U1-2)	-318	GGGGAAGTG	-326*
RNU1|+ (U1-3)	-318	GGGGAAGTG	-326*
RNU1|+ (U1-4)	-318	**GAGGAAGTG**	-326*
RNU1|- (U1-5)	-210	GAGGCAGCG	-202*
RNU1|- (U1-6)	-210	GAGGCAGCG	-202*
RNU1|- (U1-7)	-337	**GAGGAAGTG**	-345*
RNU1|- (U1-8)	-334	**GGGGAAGTG**	-342*
U1-like-1|-	-288	**GCGGAAGAG**	-280*
U1-like-2|+	-335	**GAGGAAGTG**	-343
	-288	**GCGGAAGAG**	-280*
	-147	**CCGGAAGTC**	-155
U1-like-3|+	-329	GGGGAAGTG	-337*
U1-like-5|+	-276	**GCGGAAGAG**	-268*
U1-like-6|+	-344	**GAGGAAGTG**	-352*
	-296	**GCGGAAGAG**	-289*
U1-like-7|-	-337	**GAGGAAGTG**	-345*
	-289	**GCGGAAGAG**	-281*
U1-like-8|-	-335	**GAGGAAGTG**	-343*
	-287	**GCGGAAGAG**	-279*
U1-like-9|+	-288	**GCGGAAGAG**	-280*
U2-like|-	-1172	CTGGGAGTG	-1164*
RNU3|+ (U3-1)	-267	**CCGGAAGTG**	-275*
RNU3|+ (U3-2)	-267	**CCGGAAGTG**	-275*
RNU3|- (U3-2b)	-267	**CCGGAAGTG**	-275*
RNU3|- (U3-3)	-267	**CCGGAAGTG**	-275*
RNU3|- (U3-4)	-267	**CCGGAAGTG**	-275*
RNU4ATAC|+	-464	CCGGAAATG	-472*
RNU5|+ (U5A)	-230	**CCGGAAATA**	-238
RNU5|- (U5Ds)	-306	**GCGGAAGCC**	-314
SNORD118|- (U8)	-271	GCGGAAGGG	-263*
RNU11|+ (U11)	-312	CCGGAAGGA	-304*
RNU12|+ (U12)	-303	**CCGGAAGTA**	-311*
	-255	**CCGGAAGGG**	-247
SNORD13|+ (U13)	-357	CCGGACCTA	-349*
UNKNOWN-1|+	-339	GGGGAAGTG	-347*
