# Supplementary material for: Genomic Study of RNA Polymerase II and III SNAPc-Bound Promoters Reveals a Gene Transcribed by Both Enzymes and a Broad Use of Common Activators
Source: PLoS Genet. 2012 Nov 15;8(11):e1003028. doi: 10.1371/journal.pgen.1003028 (PMC3499247; doi:10.1371/journal.pgen.1003028)
Supplement: Table S3 — Number of tags with unique and multiple matches mapped onto the genome for each ChIP_Seq experiment. (PDF) [file pgen.1003028.s014.pdf]

ChIP	Cell line	Tag length	Fragment size	Total tags sequenced*	Tags mapped allowing ≤500 matches (% of total tags sequenced)	Tags with unique match (% of total mapped tags)	Tags with 2-5 matches (% total mapped tags)	Tags with 6-10 matches (% of total mapped tags)	Tags with 10-100 matches (% of total mapped tags)	Tags with 101-500 matches (% of total mapped tags)	Total repeated tags (% of total mapped tags)
POLR3D	IMR90 hTert	36	62	35'821'593	5’305’492 (14.81)	4’016’636 (75.71)	512’919 (9.67)	180’369 (3.40)	432’009 (8.14)	136’792 (2.58)	1’262’089 (23.79)
POLR2B	IMR90 hTert	76	147	31'313'579	20’839’046 (66.55)	19’954’694 (95.76)	629’445 (3.02)	98’477 (0.47)	125’129 (0.60)	31’301 (0.15)	884’352 (4.24)
BRF2	IMR90 hTert	76	141	35'932'736	19’953’829 (55.53)	18’971’092 (95.07)	679’031 (3.40)	120’708 (0.60)	150’470 (0.75)	32’528 (0.16)	982’737 (4.93)
GTF2B	IMR90 hTert	76	134	33'534'849	22’290’367 (66.47)	21’278’323 (95.46)	706’998 (3.17)	21’021 (0.54)	150’075 (0.67)	33’950 (0.15)	1’012’044 (4.54)
SNAPC4	IMR90 hTert	76	102	22'488'382	8’486’755 (37.74)	7’984’299 (94.08)	322’151 (30.80)	66’358 (0.78)	90’921 (1.07)	16’690 (0.20)	496’120 (5.85)
SNAPC2	IMR90 hTert	76	81<br>125	17'554'105<br>17'219'539	11’327’507 (33.00)	10’652’047 (94.04)	477’172 (4.21)	81’528 (0.72)	98’497 (0.87)	12’950 (0.11)	670’147 (5.92)
SNAPC1	IMR90 hTert	76	137	23'454'787	14’680’224 (62.59)	13’988’460 (95.29)	474’514 (3.23)	83’682 (0.57)	106’142 (0.72)	27’426 (0.19)	691’764 (4.7)
SNAPC5	IMR90 hTert	76	142	34'122'704	22’858’483 (66.99)	21’810’588 (95.42)	733’165 (3.21)	125’955 (0.55)	154’550 (0.68)	34’225 (0.15)	1’047’895 (4.58)
POU2F1	HeLa	40	154	29'550'432	18’642’449 (63.09)	16’630’311 (89.21)	1’034’683 (5.55)	265’845 (1.43)	556’036 (2.98)	155’574 (0.83)	2’012’138 (10.79)
ZNF143	HeLa	38	172	31'243'832	23’973’188 (76.73)	21’203’566 (88.45)	1’384’996 (5.78)	362’217 (1.51)	773’953 (3.23)	214’048 (0.89)	2’735’214 (11.41)
GABPA	HeLa	38	172	36'171'599	26’112’641 (72.19)	23’227’432 (88.95)	1’458’350 (5.58)	378’274 (1.45)	798’559 (3.06)	215’527 (0.83)	2’850’710 (10.92)

Table S3. Number of tags with unique and repeated matches mapped onto the genome. * tags sequenced multiple times were counted only once.
