# Supplementary material for: Genomic Study of RNA Polymerase II and III SNAPc-Bound Promoters Reveals a Gene Transcribed by Both Enzymes and a Broad Use of Common Activators
Source: PLoS Genet. 2012 Nov 15;8(11):e1003028. doi: 10.1371/journal.pgen.1003028 (PMC3499247; doi:10.1371/journal.pgen.1003028)
Supplement: Text S1 — Results section describing the relationship between loci listed in Tables S1 and S2 and previously studied pol II snRNA and snoRNA genes. Methods section providing details about the stable cell lines used and about the RNase T1 protection assay. References section. (DOC) [file pgen.1003028.s015.doc]

**RESULTS**

**SNAP_c_-occupied pol II genes**

*RNU1* snRNA genes were reported to number about 30 copies per haploid genome , located on the short arm of chr 1 within p36, whereas a cluster of pseudogenes was reported on chr 1 q12-q22 . In fact, the genome assembly shows *RNU1* genes interspersed with U1-like sequences (as well as some tRNA genes, other pol III genes, and pol III gene-derived sequences) at both locations. At p36.13, there are four annotated *RNU1* snRNA genes, all of which were occupied by SNAP_c_ subunits, GTF2B, and pol II (labeled *U1-1* to *U1-4* in Table S1), and two U1-like sequences not occupied by these factors (not shown in the table). At q21.1, there are four *RNU1* snRNA genes (*U1-5* to *U1-8* in Table S1) and twenty-seven U1-like sequences, of which three (*U1-5*, *U1-6*, and *U1-8*) and eight (*U1-like-1* to *7* and *U1-like 9* in Table S1), respectively, were occupied by factors. There are, in addition, one *RNU1* gene on each chr 4, 6, and X, two on chr 14, and two U1-like sequences on chr 17 (*U1-like-10* and *-11*), of which only the last two appeared convincingly occupied by factors; the “true” *RNU1* genes on chr 14 had interrupted RPB2 peaks composed of tags with several matches in the genome, and in the other cases no peak was detected. Thus, seven of the thirteen annotated *RNU1* snRNA genes, all on chr 1, as well as eight U1-like sequences on chr 1 and two on chr 17 appeared convincingly occupied by POLR2B and other factors, and are, therefore, likely to be actively transcribed.

*RNU2* snRNA genes are organized as 10 to 20 nearly perfect 6 kb-long tandem repeats on chr 17 q21 q22 . Unfortunately, these genes are still in the “chr17_random” file of the human assembly (indicated in grey in Table S1), i.e. they could not be reliably ordered within the current sequence, and thus they were not in the reference genome used for tag alignment. However, we found one of four U2-like sequences in q21 q22 occupied by pol II and other factors; in addition, out of two annotated *RNU2* snRNA genes outside of chr 17, on chr 10 and 11, respectively, the one on chr 11 appeared occupied by GTF2B and other factors.

There are five annotated *RNU3* snRNA genes , of which three (*U3-2, U3-2b*, and *U3-4*, see Tables S1) have identical sequences to previously described human *RNU3* snRNA genes . We observed peaks on the five genes (*U3, U3-2, U3-2b, U3-3, U3-4*) as well as on a sequence annotated “*U3b2-like*”. However, as mentioned in the main text, the peaks on *U3-2, U3-2b, U3-3*, and *U3-4* were interrupted even when tags with mismatches were included, and the scores contained very few unique tags, raising the possibility that these genes are in fact very little transcribed. In contrast, *U3* and *U3b2-like* were convincingly occupied by factors.

Three human *RNU4* snRNAs have been previously described, U4A and U4B, which differed only by an additional G at the 3’end of U4A and were, therefore, hypothesized to be encoded by a single gene, and U4C . Two genes spaced by about 1.1 Kb were then cloned, one encoding U4C and the other an RNA identical to U4B but for two mismatches . We found these two *RNU4* genes, as well as the previously reported *RNU4ATAC* gene on chr 2 q14.2 , were occupied by POLR2B and other factors.

Six main types of human U5 RNA variants, U5A to U5F, have been described , as well as several subtypes, for example, a short (U5Dshort or U5Ds) and a rarer, longer form containing about ten additional 3’ nucleotides (U5Dlong), of U5D, which might be derived from the *U5Ds* gene by differential 3’ end processing . The human genome assembly contains genes corresponding to U5A, U5Ds, U5E, and U5F, two of them (*U5Ds* and *U5F*) within some 9.5 Kb of each other on chr 1. We found each of these genes, as well as four U5-like sequences (*U5A-like-1, U5B-like, U5E-like-1*, and *U5E-like-2* in Table S1), occupied by POLR2B and other factors.

There is one copy of each the *RNU7*, *SNORD118* (U8), *RNU11*, *RNU12*, and *SNORD13* (U13) RNA genes in the genome assembly, of which the *RNU11*  and *RNU12*  genes have been characterized previously, and the *SNORD118* and *SNORD13* genes correspond, except for a few mismatches, to the previously sequenced human U8 and U13 snoRNA . In each case, this copy is occupied by POLR2B and other factors. A U12 pseudogene lacking a DSE element did not display any peaks.

In addition to snRNA gene loci, we found some unannotated loci occupied by SNAP_c_ and other factors, listed at the bottom of Table S1. Among these, unknown-1 (which has 5’ and 3’ sequences similar to those of *RNU1* snRNA genes and thus a U1-like promoter) and unknown 4 are within genes predicted by geneid and transcribed in the same (chr1_338.1) and opposite (chr7_61.1) orientation, respectively; unknown-3 is just 5’ of UCSC gene CCDC80 and is transcribed in the same orientation; and unknown-7 overlaps an AluJb repeat.

**METHODS**

**Stable cell lines**

To establish the HeLa clonal cell line expressing a *RNU6* promoter-directed unstable RNA, HeLa cells were transfected by the lipofectamine (Invitrogen) method according to the manufacturer’s protocol with pDsRed-I-U6/RA.2+U6end, a derivative of pU6/RA.2+U6end-DsRed containing in addition a insulator fragment . Similarly, a clonal cell line was created by transfecting HeLa cells with pDsRed-I-U1/RA.2+U1end, which contains the human *RNU1* promoter (from -411 - +18) followed by a piece of spacer DNA unrelated to U1 and the 3′ end of the *RNU1* gene (from -5 to +92 relative to the end of the RNA-coding sequence), as well as the insulator fragment. The cells were split 48 h later and kept under G418 selection (500 μg/ml) for 21 days. Individual clones were expanded and tested for expression of the U1 or U6 reporter construct.

**RNAse T1 protection**

Five or 20 μg of RNA from cells transfected with the U1- or U6-reporter constructs was hybridized with the relevant complementary riboprobes at 55°C overnight. To analyze *RNU2* transcription, 5 μg of HeLa cell RNA was hybridized at 50°C with a complementary pre-U2 RNA riboprobe synthesized with T3 RNA polymerase from the pBSM13-preU2-T3 plasmid, which contains an *RNU2*-3’end fragment (from +123 to + 204 relative to the TSS) that overlaps the end of the mature form of U2 as well as the precursor form. The 5.8S RNA levels were measured by hybridizing 1 μg of RNA with a complementary 5.8 S RNA riboprobe synthesized with T7 RNA polymerase from the pBSKII-5.8S-T7 plasmid. T1 RNase treatment and fractionation on 6% polyacrylamide-urea gel were performed as previously described .

**REFERENCES**

57. Lund E, Dahlberg JE (1984) True genes for human U1 small nuclear RNA. Copy number, polymorphism, and methylation. J Biol Chem 259: 2013-2021.

58. Lund E, Bostock C, Robertson M, Christie S, Mitchen JL, et al. (1983) U1 small nuclear RNA genes are located on human chromosome 1 and are expressed in mouse-human hybrid cells. Mol Cell Biol 3: 2211-2220.

59. Lindgren V, Bernstein LB, Weiner AM, Francke U (1985) Human U1 small nuclear RNA pseudogenes do not map to the site of the U1 genes in 1p36 but are clustered in 1q12-q22. Mol Cell Biol 5: 2172-2180.

60. Lindgren V, Ares M, Jr., Weiner AM, Francke U (1985) Human genes for U2 small nuclear RNA map to a major adenovirus 12 modification site on chromosome 17. Nature 314: 115-116.

61. Van Arsdell SW, Weiner AM (1984) Human genes for U2 small nuclear RNA are tandemly repeated. Mol Cell Biol 4: 492-499.

62. Pavelitz T, Rusche L, Matera AG, Scharf JM, Weiner AM (1995) Concerted evolution of the tandem array encoding primate U2 snRNA occurs in situ, without changing the cytological context of the RNU2 locus. EMBO J 14: 169-177.

63. Lestrade L, Weber MJ (2006) snoRNA-LBME-db, a comprehensive database of human H/ACA and C/D box snoRNAs. Nucleic Acids Res 34: D158-162.

64. Yuan Y, Reddy R (1989) Genes for human U3 small nucleolar RNA contain highly conserved flanking sequences. Biochim Biophys Acta 1008: 14-22.

65. Krol A, Branlant C, Lazar E, Gallinaro H, Jacob M (1981) Primary and secondary structures of chicken, rat and man nuclear U4 RNAs. Homologies with U1 and U5 RNAs. Nucleic Acids Res 9: 2699-2716.

66. Bark C, Weller P, Zabielski J, Pettersson U (1986) Genes for human U4 small nuclear RNA. Gene 50: 333-344.

67. He H, Liyanarachchi S, Akagi K, Nagy R, Li J, et al. (2011) Mutations in U4atac snRNA, a component of the minor spliceosome, in the developmental disorder MOPD I. Science 332: 238-240.

68. Sontheimer EJ, Steitz JA (1992) Three novel functional variants of human U5 small nuclear RNA. Mol Cell Biol 12: 734-746.

69. Tyc K, Steitz JA (1989) U3, U8 and U13 comprise a new class of mammalian snRNPs localized in the cell nucleolus. EMBO J 8: 3113-3119.

70. Tarn WY, Yario TA, Steitz JA (1995) U12 snRNA in vertebrates: evolutionary conservation of 5' sequences implicated in splicing of pre-mRNAs containing a minor class of introns. RNA 1: 644-656.

71. Yuan CC, Zhao X, Florens L, Swanson SK, Washburn MP, et al. (2007) CHD8 associates with human Staf and contributes to efficient U6 RNA polymerase III transcription. Mol Cell Biol 27: 8729-8738.

72. Mutskov VJ, Farrell CM, Wade PA, Wolffe AP, Felsenfeld G (2002) The barrier function of an insulator couples high histone acetylation levels with specific protection of promoter DNA from methylation. Genes Dev 16: 1540-1554.

73. Lobo SM, Hernandez N (1989) A 7 bp mutation converts a human RNA polymerase II snRNA promoter into an RNA polymerase III promoter. Cell 58: 55-67.
